# A Systematic Review and Meta-analysis on the Occurrence of Biomarker Mutation in Colorectal Cancer among the Asian Population

**DOI:** 10.1155/2022/5824183

**Published:** 2022-06-23

**Authors:** Hafeez Afolabi, Salzihan Md Salleh, Zaidi Zakaria, Ch'ng Ewe Seng, Siti Norasikin Binti Mohd Nafil, Ahmad Aizat Bin Abdul Aziz, Yusuf Wada, Ahmad Irekeola

**Affiliations:** ^1^Department of General Surgery, School of Medical Sciences, Universiti Sains Malaysia Hospital, Malaysia; ^2^Department of Pathology, School of Medical Sciences, Health Campus, Universiti Sains Malaysia, Kubang Kerian, 16150 Kelantan, Malaysia; ^3^School of Medical Sciences, Hospital Universiti Sains Malaysia HUSM, Universiti Sains Malaysia USM, Kubang Kerian, 16150 Kelantan, Malaysia; ^4^Department of Medical Microbiology and Parasitology, School of Medical Sciences, Health Campus, Universiti Sains Malaysia, Kubang Kerian, 16150 Kelantan, Malaysia

## Abstract

Globally, colorectal carcinoma (CRC) is the third most common cancer and the third major cause of cancer-related death in both sexes. KRAS and BRAF mutations are almost mutually exclusively involved in the pathogenesis of CRC. Both are major culprits in treatment failure and poor prognosis for CRC. *Method*. A systematic review and meta-analysis of various research was done following the Preferred Reporting Items for Systematic Reviews and Meta-Analysis (PRISMA) guidelines. This trial is registered with PROSPERO CRD42021256452. The initial search included 646 articles; after the removal of noneligible studies, a total of 88 studies was finally selected. Data analysis was carried out using OpenMeta Analyst and Comprehensive Meta-Analysis 3.0 (CMA 3.0) software to investigate the prevalence of KRAS and BRAF mutations among patients with CRC in Asia. *Results*. The meta-analysis comprises of 25,525 sample sizes from Asia with most being male 15,743/25525 (61.7%). Overall prevalence of KRAS mutations was (59/88) 36.3% (95% CI: 34.5-38.2) with *I*^2^ = 85.54% (*P* value < 0.001). In 43/59 studies, frequency of KRAS mutations was majorly in codon 12 (76.6% (95% CI: 74.2–78.0)) and less in codon 13 (21.0% (95% CI: 19.1-23.0)). Overall prevalence of BRAF mutations was 5.6% (95% CI: 3.9-8.0) with *I*^2^ = 94.00% (*P* value < 0.001). When stratified according to location, a higher prevalence was observed in Indonesia (71.8%) while Pakistan has the lowest (13.5%). *Conclusion*. Total prevalence of KRAS and BRAF mutations in CRC was 36.6% and 5.6%, respectively, and the results conformed with several published studies on KRAS and BRAF mutations.

## 1. Introduction

Globally, cancer is a serious medical burden, and it is one of the main causes of death and morbidity throughout the world [1, 2]. With more than 1.8 million new CRC diagnoses and 0.86 million deaths globally in 2018 [3], colorectal carcinoma (CRC) is the third most common cancer and the third major cause of cancer-related death in both sexes [4]. The occurrence of CRC differs globally; the overall highest incidence rates of CRC may be seen in the United States, Canada, Europe, and Australia, whereas the lowest rates can be found in South-Central Asia and Africa [2]. However, the prevalence is rising exponentially in Asia [5], especially as the number of new cases of CRC is rapidly growing in Asia-Pacific thus, accounting for more than half of all new cases diagnosed globally [5, 6]. CRC prevalence rates vary due to several factors such as ethnicity, genetics, regions, and lifestyles. It is reported to be 38 percent among Caucasians, 40 percent among Asians, and just 21 percent among Africans [6]. Pathogenesis of CRC involves gene mutation, mostly involving the MAPK-ERK cascade, for which the KRAS and BRAF are exclusively involved.

Nation-wise, the prevalence of KRAS and BRAF mutation varies regionally, and this is majorly due to genetic changes from heterogeneously related races [6]. From the World Health Organization (WHO) regional grouping, the prevalence of KRAS mutation among constituting nations with CRC is 30.23%, 35.12%, 31.83%, 33.17%, and 32.64% for the EMRO, EURO, PAHO, SEARO, and WAPRO, respectively, [5]. Colorectal carcinogenesis is a multisignalling process involving four major pathways: the Wnt-*β* catenin pathway, MAPK/ERK pathway, PI3K/Akt pathway, and p53 pathway. Each pathway involves several sequential genetic modifications, such as chromosomal anomaly, gene mutations, and/or epigenetic changes, that turn normal colonic epithelium into colorectal cancer [7] [8]. Like the KRAS gene, BRAF is also part of the Ras family that targets the RAS/RAF/MEK/ERK pathway; together, they both account for 7-25% and 5-20% of all cancers as well as 30-45% and 8–10% of CRC for both KRAS and BRAF, respectively [8, 9]. Mutations in KRAS and BRAF are almost mutually exclusive. The detection or testing for KRAS and BRAF gene mutation presents a blueprint and change to standard diagnostic guidelines for inpatient care and creates a major development in early decision-making in personalizing cancer care. The identification of this mutation would be crucial for the prognosis of CRC patients. Early diagnosis and treatment will improve patients' standard of health, increase their chances of survival, and lower morbidity and mortality. The poor prognosis of metastatic CRC has fuelled continued efforts to identify therapeutic options that will improve patient outcomes via detailed gene profiling such as in KRAS and BRAF mutations.

Ras proteins are tiny guanosine triphosphatases (GTPases) to which the KRAS and BRAF genes belong. Through the GTPase cascade, they govern a variety of intracellular activities such as proliferation, differentiation, immune response, and survival rate [10]. The understanding of genetic alterations (such as in KRAS and BRAF mutations) in metastatic CRC (mCRC) via the use of gene profiling can be a catch point in explaining the gene's resistance to antiepidermal growth factor receptor (EGFR) antibody management [11] and as a prognostic predictor in arresting the progress of CRC [12] [8, 13]. Both KRAS and BRAF are downstream EGFR oncogenes, for which their mutations can activate EGF receptor signalling in cancer cells and are linked with poor prognosis in the CRC. Hence, certain aberrations or mutations that have a well-established prognostic and predictive blueprint in CRC are now regularly examined as a component of clinical therapy [14]. Through this study, the authors intend to determine the prevalence of KRAS and BRAF gene mutations in CRC and whether the prevalence of mutated KRAS and mutated BRAF genes in colorectal carcinoma differs among patients in Asia via literature review and meta-analysis to provide a very accurate KRAS and BRAF mutation estimates.

## 2. Materials and Methods

The present study is a systematic review and meta-analysis of various researched and published papers that were carried out following the guidelines of the Preferred Reporting Items for Systematic Reviews and Meta-Analysis (PRISMA) [15]. The study protocol was registered with PROSPERO (registration number: CRD42021256452).

### 2.1. Literature Search and Selection Criteria

**Figure 1:**
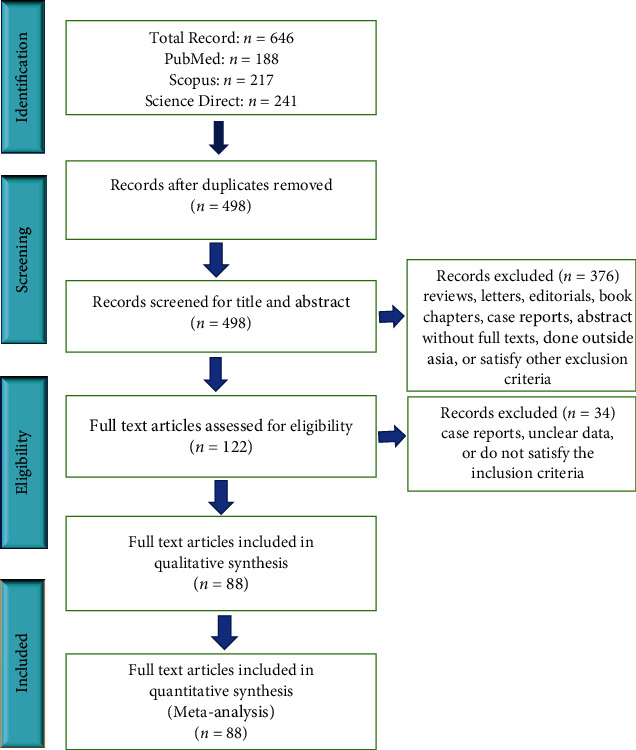
Summary of article identification and selection process.

In the study, articles were retrieved from three electronic databases (PubMed, Scopus, and ScienceDirect); the eligible studies were searched and assessed using a combination of relevant keywords: (“colorectal cancer” OR “colon cancer” OR “metastatic colon cancer” OR “metastatic colorectal cancer” OR “CRC” OR “Rectum”) and (“BRAF” OR “BRAF” OR “c-BRAF” OR “KRAS” OR “K-RAS” OR “c-KRAS”) and (“Asia”). The full details of the search strategies for this study are in the supplementary search strategic file. A comprehensive search for the most relevant studies was carried out by combing through titles, keywords, and abstracts of various papers. The initial search included 646 articles (Figure 1) which were performed on 10 April 2021 via the EndNote X9 software; references of all assessed studies were exported to the software after which, duplications were then removed. The inclusion criteria for the studies selected for this meta-analysis include cross-sectional studies, cohort studies, or case series carried out to investigate the frequency of KRAS or BRAF gene mutations in colorectal cancer patients in Asia. Also included are studies on KRAS and BRAF gene mutations from fresh frozen, formalin-fixed paraffin-embedded (FFPE), or biopsied colorectal carcinoma specimens. Also, KRAS and BRAF studies involving more than one sample size and all related papers published at valid international summits were included. No limit is set on methods for determining gene mutations. The exclusion criteria include (1) studies not associated with frequency of KRAS and BRAF gene mutations, (2) studies that investigated just one of either codon 12 or codon 13 of KRAS gene mutation, (3) reviews and case reports, (4) KRAS and BRAF gene mutations that are related to cell lines and animal studies, and (5) studies that investigated BRAF gene mutation through KRAS-positive patients [16]. All authors were involved in the study screening, selection, and assessment criteria. Two authors (A.H.A. and A.A.I.) independently screened the articles based on title and abstract. This was proceeded by the assessment of the full texts. Any discords during the screening process were resolved by discussion with other authors in the study.

### 2.2. Data Extraction and Quality Assessment

The data extraction was carried out by using an Excel spreadsheet. Two reviewers (H.A.A. and A.A.I.) independently vetted the titles and abstracts and extracted crucial information required; study I.D, publication year, period and design, gender, data on the mutation of KRAS, and BRAF prevalence among patients diagnosed with colorectal cancer in Asia were diligently extracted. Any inconsistencies were handled through conversation with a third reviewer (S.M.S) to prevent any sort of prejudice, and any discrepancies were sorted out via dialogue involving other reviewers to avoid any kind of bias. The quality of the methodological approach for the studies included was appraised independently by two authors (A.H.A. and Y.W.) via the Joanna Briggs Institute (JBI) critical appraisal checklist for prevalence data [17] (Supplementary JBI file). A score of 1 for “yes” and 0 for other parameters was allotted to obtain a sum quality score that ranges between 0 and 9. Studies with a final score of 7–9 were chosen to be of desirable quality. The studies within the latter acceptable score range were included in the data extraction phase of the meta-analysis.

### 2.3. Data Synthesis and Analysis

Data analysis was carried out via the use of OpenMeta Analyst and Comprehensive Meta-Analysis 3.0 (CMA 3.0) software [18]. The prevalence of KRAS and BRAF gene mutations among patients with colorectal carcinoma in Asia was calculated, and subgroup analysis was also carried out on location, tumour stage, tumour grade, and period of study. A random-effect model through the DerSimonian-Laird method of the meta-analysis was employed to obtain the pooled estimates of the reported KRAS and BRAF gene mutation cases. Further, to ascertain the study quality, possible publication bias was scrutinized by creating a funnel plot. The asymmetry of the plot was further investigated via Egger's regression test [19]. The heterogeneities of study-level estimates were determined by Cochran's *Q* test and quantified using *I*^2^ statistics. *I*^2^ values of 25%, 50%, and 75% were considered low, moderate, and high heterogeneities, respectively [20]. For all tests, a *P* value < 0.001 was labelled statistically significant.

## 3. Result

This section is divided into subheadings to provide a concise and precise description of the experimental results and their interpretation, as well as the experimental conclusions that can be drawn from the outcomes.

### 3.1. Search Results and Study Selection

A total of 646 records were obtained by searching three electronic databases. After eliminating duplications and studies that do not favour the inclusion criteria, the remaining 498 were screened via titles and abstracts and by further excluding 261 records that satisfied the exclusion criteria and another 115 records that were done outside Asia; then, 122 records were left. Upon further scrutiny, another 34 records that did not merit the inclusion criteria as depicted in Figure 1 above were excluded. Finally, a total of 88 unique records were confirmed eligible to be included in the meta-analysis. Among these later 88 eligible studies, 59 reported on KRAS gene mutation, and 29 reported on BRAF gene mutation. Thus, a total of 88 studies were selected for this meta-analysis.

### 3.2. Characteristics of the Eligible Studies

The characteristics of studies on KRAS and BRAF mutations are illustrated in Tables 1 and 2, respectively. The meta-analysis study comprises of 25,525 sample sizes; all studies were from the Asian region with the male patient being most of the total participants, 15,743 out of 25525 (61.7%). The comprehensive characteristics of the included studies are illustrated in Table 1.

### 3.3. Prevalence of KRAS Mutations in CRC Patients

**Figure 2:**
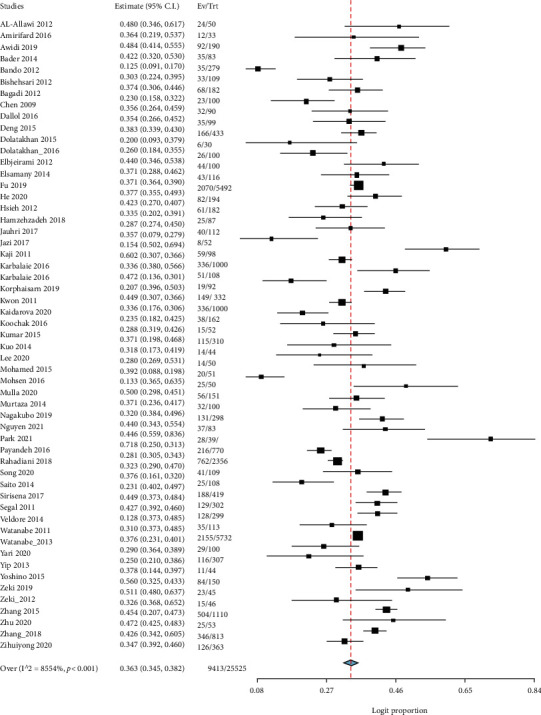
A forest plot for the prevalence of KRAS mutation in Asian CRC patients.

The prevalence of KRAS gene mutation illustrated in the 59 studies included in the meta-analysis involves a total of 25525 patients. Among the studies, the highest frequency of KRAS mutations reported by Rahadiani et al. [57] was 71.8% (95% CI: 55.9-83.6) (38), and the lowest frequency of KRAS mutations was reported by Bakarman and AlGarni [2] was at a rate of 12.5% (95% CI: 9.1-17.0) (37). Using the random-effect model, the overall prevalence of KRAS mutations among Asians was 36.3% (95% CI: 34.5-38.2) with *I*^2^ = 85.54% and (*P* value < 0.001) (Figure 2). Furthermore, in 43 out of 59 studies, the frequency of KRAS gene mutations was reported in codon 12 and codon 13. The prevalence of mutated codons across all KRAS mutations could be seen in supplementary figures SF1 and SF2. Codon 12 and codon 13 mutations were discovered in the populations to be 76.6% (95% CI: 74.2-78.8) and 21.0% (95% CI: 19.1-23.0), respectively (Supplementary figures SF1 and SF2).

### 3.4. Prevalence of KRAS Gene Mutation in Colorectal Cancer Stratified by Study Location and Period of Study

To determine the prevalence of KRAS mutation in CRC patients from various regions in Asia, a subgroup meta-analysis was undertaken. Data were available for nineteen locations from the listed studies, with the largest number of studies coming from Iran (*n*: 12) (Table 3; Supplementary Figure SF3).

The country of Indonesia had the highest prevalence rates projection at 71.8% (95% CI: 55.9–83.6), while Pakistan had the lowest estimate of 13% (95% CI: 8.8-19.8) (Table 3; Supplementary Figure SF3). Greater heterogeneity was found in studies from Saudi Arabia, China, South Korea, and India (*I*^2^ = 91.95%, 91.21%, 87.18%, and 83.77%), respectively (*P* value < 0.001), which may have added to the overall heterogeneity found in the outcome.

On the period of study, studies done “after 2010” had the highest number of studies (28) during the study period (Table 3; Supplementary Figure SF4) with the highest KRAS prevalence at 39.9% (95% CI: 37.3–42.5), while those done “2010 and below” had KRAS prevalence at 32.3% (95% CI: 28.8–36.0), respectively (*P* value < 0.001).

On the tumour stage, KRAS mutation was reported highest in the late stage at 67.9% (95% CI: 59.3–75.5), while on location, the colon recorded the highest KRAS mutation of 61.2% (95% CI: 55.1–67.0). On the grading of KRAS mutation in CRC, “Moderate grading” recorded the highest KRAS mutation of 51.8% (95% CI: 42.9–61.2) (Table 3; Supplementary Figure SF6, 8, and 10, respectively).

### 3.5. Prevalence of BRAF Gene Mutation of Patients with Colorectal Cancer Stratified by Forest Plot for BRAF

**Figure 3:**
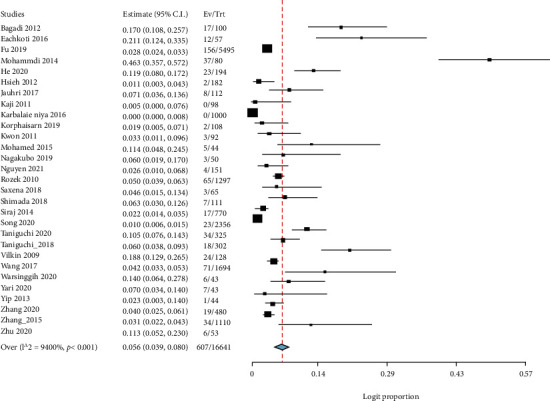
Forest plot for BRAF.

The prevalence of BRAF gene mutations in colorectal patients was investigated using the random-effect model. In the 29 (607 patients) out of 88 studies that reported BRAF prevalence, the highest prevalence was reported by Jauhri et al. [39] at 7.1% (95% CI: 3.6–13.6) and Yari et al. [67] at 7.0% (95% CI: 3.4–14.0), respectively. The least BRAF gene mutation was reported by Hsieh et al. [37] at 1.1% (95% CI: 0.3–4.3). In 2 out of the 29 studies, Kaji et al. [41] and Niya et al. [42] reported no BRAF mutation: 0% (95% CI: 0.0–7.6) and 0% (95% CI: 0.0–0.8), respectively. The overall prevalence of BRAF gene mutations was 5.6% (95% CI: 3.9-8.0) with *I*^2^ = 94.00% and (*P* value < 0.001) (Figure 3). In all the studies (29 out of 88), the screening of BRAF gene mutations was based on the detection of BRAF-V600E mutation.

### 3.6. Subgroup Analysis of the Prevalence of BRAF Gene Mutation in Patients with Colorectal Cancer Stratified by Study Location and Period of Study Conduct

To determine the prevalence of BRAF in colorectal cancer CRC patients from various regions in Asia, a subgroup meta-analysis was undertaken. Data were available for fourteen locations from the listed studies, with the largest number of studies coming from China (*n*: 7) (Table 4; Supplementary Figure SF12).

India had the highest prevalence rate projection at 11.7% (95% CI: 6.2 – 21.0), while Taiwan had the lowest estimate of 1.1% (95% CI: 0.3-4.3) (Table 4; Supplementary Figure SF12). Greater heterogeneity was found in studies from China and Iran (*I*^2^ = 91.21% and 96.16%), respectively (*P* value < 0.001), which may have added to the overall heterogeneity found.

On the period of study, studies done “after 2010” had the highest number of studies (17) during the study period (Table 4; Supplementary Figure SF13) with the highest BRAF gene mutation prevalence at 5.4% (95% CI: 3.7–7.7), while those done “2010 and below” had BRAF mutation prevalence at 5.6% (95% CI: 2.0–14.6), respectively (*P* value < 0.001).

On the tumour stage, BRAF mutation was reported highest in the “late stage” at 59.9% (95% CI: 48.2–70.7), while on location, “colon” recorded the highest BRAF mutation of 67.9% (95% CI: 37.3–42.5). On the grading of BRAF mutation in CRC, “moderate grading” recorded the highest BRAF mutation of 56.3% (95% CI: 43.3–68.6) (Table 4; Supplementary Figures SF15, 17, and 19).

### 3.7. Analyses of Sensitivity and Publication Bias

**Figure 4:**
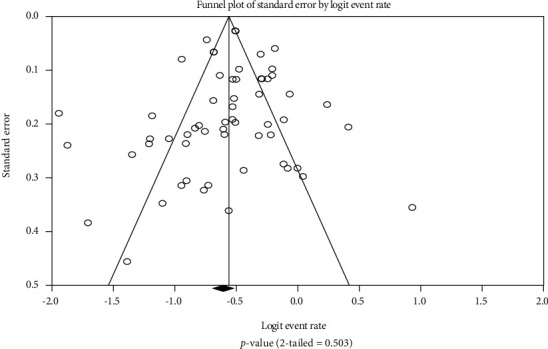
KRAS funnel plot. *P* value: 2-*tailed* = 0.503.

**Figure 5:**
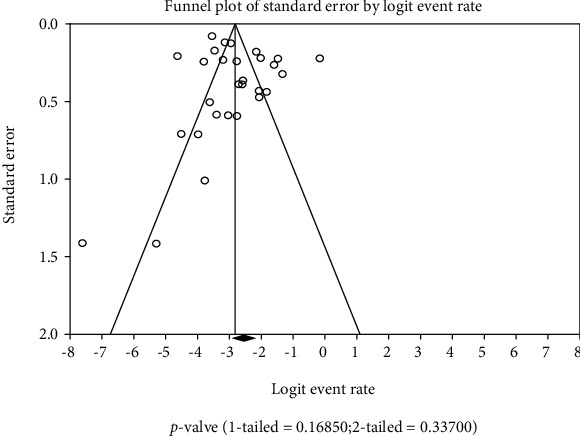
BRAF funnel plot. *P* value: 1-*tailed* = 0.16850; 2-*tailed* = 0.33700.

A funnel plot of random effects was generated to observe evidence of publication bias among the studies reporting KRAS gene mutation (Figure 4) and BRAF gene mutation (Figure 5) among Asian CRC patients. There was no clear evidence of publication bias in both KRAS and BRAF mutation studies.

## 4. Discussion

Several research today showed that mutations in the RAS family of genes especially the KRAS are linked to around a third of all malignancies; however, the incidence of the gene mutations varies greatly depending on the kind of cancer: often seen to be 40% in colorectal cancer, 15-20% in non-small-cell lung cancer, and 95% in pancreas carcinoma [44]. Only a few individuals diagnosed with colorectal cancer would be opportune to receive curative surgery if detected early because, at the time of consult with the surgeon, it is already in the late stage wherein the prognosis is poor. More so, the illness involves no specific early presenting features, and the long-term disease period is usually associated with probable organ metastases [86, 88]. Also, because colorectal cancer is thought to grow progressively over time due to the buildup of genetic abnormalities, the threat of reoccurrence and mortality from colorectal cancer is significantly linked to the stage of the disease at diagnosis [86]. Although there is a tremendous advance in the CRC treatment via the use of cytotoxic agents, i.e., monoclonal antibodies to targeted therapy such as on EGF receptor [78], CRC still poses a significant threat to life as KRAS gene mutation is reported as a major cause of treatment failure in cancer therapy [89].

Colorectal cancer (CRC) is the third most frequent cancer in the world, with 2.0 million new cases in 2020, accounting for 11% of all new cancer cases [90]. It was estimated as 1.9 million of all new cases and 880,000 deaths in 2018 [91]. The incidence and mortality rates of colorectal cancer (CRC) differ significantly around the globe, i.e., differs in various regions. From a total of 646 eligible papers that were filtered in this study, 88 studies were finally selected to investigate the prevalence of KRAS and BRAF gene mutations in this analysis. During this analysis, approximately 115 articles reporting KRAS and BRAF gene mutations in CRC outside Asia were identified, but they were, however, excluded because they did not fulfill the study's inclusion criteria. This plethora of articles discovered spanned almost every corner of the world. Balschun et al. [92] documented the prevalence case of KRAS and BRAF in German patients in Europe. Di Fiore et al. [93] reported the first instance of KRAS and BRAF mutations in CRC in the United Kingdom. Raskin et al. [94] and Osasan [95] studies were done in Africa. Altogether, these illustrated the different prevalence of KRAS mutation existence in CRC around the globe.

In this study, the prevalence of KRAS and BRAF mutations was investigated in 88 studies involving 25,527 CRC patients from various countries in Asia; the overall prevalence of KRAS gene mutations was found to be 36.3% (95% CI: 34.5-38.2). KRAS gene mutations are a well-investigated mutation in several carcinomas such as melanoma [96], non-small-cell lung carcinoma [97], colorectal carcinoma [98, 99], and papillary thyroid cancer [100]. KRAS gene mutations, which function as an active oncogene, are found in 35 to 45 percent of CRC cases globally [50, 101–103]. The findings of these investigations corroborate our study's outcome that approximately thirty-six percent of the patients have KRAS gene mutation. This prevalence rate was similar to data reported from the US (35.7% [104] and 31% [105]), China (32%) [106], Japan (33.5%) [107], Taiwan (33.5%) [37], Russia (35.9%) [108], France (33.8) [109], the United Kingdom (36.9%) [110], and Brazil (36%) [111], although KRAS prevalence was reported to slightly differ from some published data from Germany (41%) [112], Italy (62.2%, 43%, and 43%) [113–115], Turkey (44%) [116], Morocco (24%) [117], and Egypt (11% and18.4%) [118, 119]. These latter differences could be associated with various factors such as race, lifestyle, period, and means of sample collection and geographical locations.

The dynamic of gene expression patterns on gender and age was investigated by some studies as a possible risk for developing CRC [120, 121]. In this study, the age of the participants was also taken into consideration, the bulk of the recruited participants were adults, with most of them being over 50 years old, implying that KRAS gene mutation predominates in adult CRC. It was indeed as anticipated, given that older age has hitherto been identified as a health risk for CRC in numerous investigations [122]. Although data on gender were not reported for some studies in the included studies for this analysis, CRC was found to be more common in male patients (60.7%) than female patients (39.3%). This information points to the importance of gender predilection in the occurrence of CRC which is consistent with findings from other studies around the world [123, 124]. On the location of the tumour, the cancer was mostly found in the colon (82.1%) which is a similar finding in several studies [39], probably because the patient would present at the latter stage of cancer [125].

Although human scientific knowledge has greatly advanced compared to decades ago, however, our study found that the prevalence of KRAS gene mutation was higher among patients screened “after 2010,” 36.7% (95% CI: 34.6-38.8), than when compared to those screened “before 2010,” 32.3% (95% CI: 28.8-36.0), probably due to medical advances and more medical screening [126]. During the shedding of tumour cells or apoptosis, small DNA fragments flow into the blood system, leading to the detection of this ctDNA mutation in almost all cancer types and in the late stages of the tumour or the malignancy, hence, more frequency of the DNA mutation detection on screening. Another reason could range from lifestyle evolution to dietary choice, synergically working together to modify our body biocomposition and genetic make-up [127]. The late-stage (stages 3 and 4) recorded more KRAS gene mutation (68%) than the early stage (30%) but this could be associated with discrepancies in the time of consultation and stages of the tumour at the time of recruitment of the patients for the included studies, as the majority of the mean age reported by the studies was in 5th or 6th decade of life and because most of the patient would have distant metastases at the period of diagnosis.

On the location of KRAS and BRAF gene mutations, the colon (61% and 68%), respectively, was the most recorded mutation site which on the contrary is the rectum [128]; however, this is as expected as the main physiological function of the intestinal lumen of the colon includes water absorption and stool storage. Therefore, the contents contained inside the colon are relatively desiccated which is the tumour-conducive condition for gene mutation detection [129, 130]. In this present research, the majority of KRAS mutations occurred in codon 12, 76.6% (95% CI: 74.2-78.8), than in codon 13, 21.0% (95% CI: 19.1-23.0). These findings are comparable to those of previous research [105, 116, 117]. For example, in a Belgian research, 36.3 percent of people had KRAS mutations, with 91 percent of mutations in codon 12 [131]. Another study published in Dobre et al. [132] found that KRAS mutations in codons 12 and 13 were found in 79.3 percent and 19.7 percent of people, respectively. A similar study in Brazil reported that 87% of KRAS mutations were in codon 12 and 13% in codon 13. However, research in the Greek population found that KRAS mutations at codon 12 are uncommon (29.3%) [133]. Only 3 studies of the colorectal cancer patients in our analysis had a KRAS codon 61 mutations [26, 67, 68] which is not surprising given that the majority of KRAS mutations reported in human tumours are in codon 12, with mutations in codons 13 and 61 accounting for only 1.7-9 percent [27].

BRAF is also a member of the RAF gene subfamily that, like KRAS, performs its function in the EGFR downstream cascade, but their mutations are less frequent than the KRAS gene mutations. Among the BRAF gene, BRAFV600E mutation is the most prevailing [134], and in this present study, BRAFV600E mutation is used to examine the prevalence of BRAF gene mutation in CRC. The frequency of BRAF mutation varies globally, approximately 1.1–25% [16, 49, 135–139]. The prevalence of BRAF mutation obtained in this study was 5.6% (95% CI: 3.9-8.0), and this is conforming with the several existing findings, i.e., 1.1 to 5.8% in Asian studies and 5–21% in western studies [112, 131, 134, 140–142]. Another reason for these prevalence similarities could be associated with genetic homogeneity as the studies involve certain regions, and their lifestyles and diet are almost similar [143]. BRAF-activating mutations are frequently exclusive with KRAS mutations, accounting for 5–15% of mCRC cases, and are linked to a poor prognosis in stages II, III, and IV [144]. This mutation causes a constant stimulation of the mitogen-activating protein kinase MAPK pathway, which controls the transcriptase activity of regulatory genes in the cell cycle by modulating cell growth stimuli, a nonfunctioning condition that predisposes to cancerous growth [145].

This study possesses several merits and strengths. First, to the best of the author's knowledge, it has been the first systematic review and meta-analysis carried out on the prevalence of KRAS and BRAF mutations among Asians with CRC. Also, a well-detailed and comprehensive search strategy ensures that elaborate all-inclusive papers are included, thus leading to a very large population size of 25,525. This also ensures high confidence in the outcomes obtained since the included studies were of high methodology quality. However, this analysis was not without some limitations, with many linked to the data from the literature of the included studies such as small sample size, incomplete reports on sex, mean age, period of study conduction, differentiation, and location of the tumour, and lastly, mutation screening was done just for the BRAFV600E. All these parameters/characteristics that would be crucial in upholding the study appraisal were not reported in some of the studies analysed in this meta-analysis, thus accounting to some of the heterogeneity seen in the studies.

## 5. Conclusions

This systematic review and meta-analysis study, which to the best of our knowledge, is the first to report on the prevalence of KRAS (36.6%) and BRAF (5.6%) mutations in CRC patients in Asia. The result showed that the rate of KRAS and BRAF gene mutations in CRC among Asians is rising. The adult age was more associated with CRC prevalence, and the males have increase fold and poorer outcome than their female counterparts. Despite some limitations, the meta-analysis yielded impressive results. The total prevalence of KRAS and BRAF mutations, 36.6% and 5.6%, respectively, differs in various countries in Asia according to this meta-analysis. Furthermore, when the findings of this study were compared to those of other studies, it was discovered that the prevalence of these mutations obtained in our analysis conformed with them.

## Figures and Tables

**Table 1:** Major characteristics of the prevalence of KRAS screening studies that were included in the meta-analysis.

S/N	Author	Year	Location	Male, *n* (%)	Age	Sample size	Tumour stage (early stage)^∗^	Tumour stage (late stage)^∗^	Tumour location (colon)^∗^	Tumour location (rectum)^∗^	Tumour grade (poorly differentiated)^∗^	Tumour grade (moderately differentiated)^∗^	Tumour grade (well-differentiated)^∗^	Method	Total KRAS mutation (%)	KRAS (codon 12) %	KRAS (codon 13) %
1	Al-Allawi et al., 2012 [21]	2012	Iraq	54	55.4 ± 15.25	50	50	50	44	56	18	62	20	Sequencing	48	91.7	8.3
2	Amirifard et al., 2016 [22]	2016	Iran	79	51.5 ± 12.6	33	0	100	55	45	9	21.2	69.8	Sequencing	36.4	91.7	8.3
3	Awidi et al., 2019 [23]	2019	Jordan	60	58 (19-83)	190	NR	NR	97.4	2.63	NR	NR	NR	Sequencing	48.4	81.5	17.4
4	Bader and Ismail, 2014 [24]	2014	Saudi Arabia	58	55 (26-90)	83	13.3	86.7	76	24	9.6	82	8.4	Sequencing	42.2	88.6	11.4
5	Bakarman and AlGarni, 2019a [2]	2019	Saudi Arabia	56	57 ± 13	279	34	56.6	59.5	40.5	6.5	73.8	5.7	Sequencing	12.5	NR	NR
6	Bando et al., 2012 [25]	2012	Japan	60	NR	109	NR	NR	69.7	30.2	NR	NR	NR	Sequencing	30.3	78.8	21.2
7	Bishehsari et al., 2006 [26]	2006	Iran	57	NR	182	NR	NR	71	29	NR	NR	NR	Sequencing	37.4	66.2	32.4
8	Bagadi et al., 2012 [27]	2012	India	74	56 (23-93)	100	22.5	77.5	78	22	NR	NR	NR	Sequencing	23	87	13
9	Chen et al., 2009 [28]	2009	Taiwan	54	25-90	90	NR	NR	68.9	31.1	NR	NR	NR	Sequencing	35.6	75	25
10	Dallol et al., 2016 [29]	2016	Saudi Arabia	59	NR	99	NR	NR	72.7	27.3	10.1	61.6	16.2	HTT-sequencing	35.4	NR	NR
11	Deng et al., 2015 [30]	2015	China	59	NR	433	50.3	49.7	73.9	26.1	21.2	49.2	21.9	Sequencing	38.3	74.1	25.9
12	Dolatkhah et al., 2015 [31]	2015	Iran	77	77.6 (27–90)	30	36.7	46.7	NR	NR	6.7	26.7	50	Sequencing	20	NR	NR
13	Dolatkhah et al., 2016 [32]	2016	Iran	65	61.9 ± 15.34	100	37	29	72	28	3	22	49	Sequencing	26	61.5	34.6
14	Elbjeirami and Sughayer, 2012 [33]	2012	Jordan	55	55 (22-74)	100	5	95	78	22	NR	NR	NR	Sequencing	44	88.6	11.4
15	Elsamany et al., 2014 [34]	2014	Saudi Arabia	54	NR	116	23.3	76.7	67.8	32.2	29.3	58.7	12	Sequencing	37.1	NR	NR
16	Fu et al., 2019 [35]	2019	China	60	60 (14-96)	5495	NR	NR	50.1	49.9	6.5	71.3	24.4	HRMS	37.7	75.1	22.2
17	He et al., 2020 [36]	2020	China	62	59 (26-83)	194	4.1	83	72.6	27.3	45.9	NR	NR	Sequencing	42.3	63.4	17.1
18	Hsieh et al., 2012 [37]	2012	Taiwan	NR	NR	182	NR	NR	NR	NR	NR	NR	NR	Sequencing	33.5	NR	NR
19	Hamzehzadeh et al., 2018 [38]	2018	Iran	59	57 (27-86)	87	NR	NR	87.3	12.6	4.6	77	18.3	Sequencing	28.7	72	28
20	Jauhri et al., 2017a [39]	2017	India	61	NR	112	23.2	76.8	82.1	17.9	NR	NR	NR	Sequencing	35.7	67.5	17.5
21	Jazi et al., 2017 [40]	2017	Iran	56	61.2 ± 9.13	52	55.8	28.8	28.8	71.2	15.4	42.3	23.1	Sequencing	15.4	75	25
22	Kaji et al., 2011 [41]	2011	Japan	35	68.9 ± 9.8	98	NR	NR	NR	NR	NR	NR	NR	NR	60.2	71.2	23.7
23	Karbalaie Niya et al., 2016 [42]	2016	Iran	57	NR	1000	NR	NR	NR	NR	16.4	38.4	43.9	HRMS	33.6	85.1	14.9
24	Korphaisarn et al., 2019 [14]	2019	Thailand	57	64 (30-89)	108	24.1	75.9	82.4	17.6	6.5	86.1	4.6	PNAM-PCR	47.2	NR	NR
25	Kwon et al., 2011 [43]	2011	South Korea	60	54 ± 12.33	92	0	100	56.5	43.5	15.2	67.4	12	PNAM/PCR/S	20.7	NR	NR
26	Kaidarova et al., 2020 [44]	2020	Kazakhstan	45	56.4 + 10.5	332	NR	NR	NR	NR	NR	NR	NR	PCR	44.9	80.5	19.5
27	Koochak et al., 2016 [16]	2016	Iran	57	NR	1000	0	100	NR	NR	16.4	38.4	43.9	HRMA/P	33.6	85.1	14.9
28	Kumar et al., 2015 [45]	2015	Oman	59	56 (18–80)	162	22.8	75.3	29.6	70.4	16.7	77.2	5.6	IHC	23.5	NR	NR
29	Kuo et al., 2014 [46]	2014	Taiwan	54	63.2 (30-88)	52	9.6	90.4	67.3	30.8	5.8	84.6	3.8	PNA-M/PCR	28.8	66.7	33.3
30	Lee et al., 2020 [47]	2020	South Korea	43	62 (27-88)	310	23.6	74.9	NR	NR	NR	NR	NR	Sequencing	37.1	76.5	23.5
31	Mohamed Suhaimi et al., 2015 [48]	2015	Singapore	54	58.5 (26-74)	44	50	40.9	54.5	45.5	NR	NR	NR	HRM-S	31.8	NR	NR
32	Mohsen et al., 2016 [49]	2016	Iran	70	62.17 ± 14.18	50	NR	NR	74	26	NR	36	30	Sequencing	28	71.4	28.6
33	Mulla et al., 2020 [50]	2020	Saudi Arabia	51	60 (28-91)	51	35.3	64.7	NR	NR	3.9	84.3	11.8	Histopathology	39.2	75	20
34	Murtaza et al., 2014 [51]	2014	Pakistan	64	NR	150	12	88	48	52	38.7	26.7	34.7	Sequencing	13.3	60	35
35	Nagakubo et al., 2019 [52]	2019	Japan	NR	NR	50	NR	NR	NR	NR	NR	NR	NR	Sanger sequencing	50	68	20
36	Nguyen et al., 2021 [53]	2021	Vietnam	56	59.94 ± 12.36	151	76.2	19.2	68.9	31.1	10.2	36.2	53.2	Sanger sequencing	37.1	55.4	44.6
37	Omidifar et al., 2015 [54]	2015	Iran	55	59.08 ± 15.55	100	NR	NR	NR	NR	NR	NR	NR	Sequencing	32	71.9	25
38	Park et al., 2021 [55]	2021	South Korea	61	23–93	298	55.4	44.6	72.8	25.2	6	72.5	21.5	Sequencing	44	NR	NR
39	Payandeh et al., 2016 [56]	2016	Iran	61	57.7 ± 13.0	83	0	100	61.4	38.6	7.3	32.5	60.2	HRM/AS/PCR/P	44.6	81.1	18.9
40	Rahadiani et al., 2018 [57]	2018	Indonesia	55	NR	39	NR	NR	NR	NR	2.6	NR	69.2	RT-PCR	71.8	75	17.9
41	Siraj et al., 2014 [58]	2014	Saudi Arabia	51	NR	770	44.5	49.2	NR	NR	12.1	76.6	9.6	PCR/sequencing	28.1	70.4	29.6
42	Song et al., 2020 [59]	2020	China	61	NR	2356	44.8	45.2	NR	NR	18.7	NR	81.3	Sequencing	32.3	NR	NR
43	Saito et al., 2014 [60]	2014	Japan	58	63.5 (20-82)	109	NR	NR	NR	NR	NR	NR	NR	ARMS/S-assay/DS	37.6	68.3	31.7
44	Sirisena et al., 2017 [61]	2017	Sri Lanka	63	61 (29-85)	108	NR	NR	NR	NR	NR	NR	NR	PCR/sequencing	23.1	60	40
45	Segal et al., 2011 [62]	2011	Israel	46	NR	419	NR	NR	NR	NR	NR	NR	NR	Sequencing	44.9	82.4	17.6
46	Taniguchi, H., et al. 2018 [63]	2018	Japan	59	64 (26-89)	302	26.8	73.2	53	47	9.6	NR	NR	Sequencing	42.7	69.8	15.5
47	Veldore et al., 2014 [64]	2014	India	65	55.9 ± 12.8	299	NR	NR	70.2	29.8	7.02	13	79.9	RT/PCR/S	42.8	92.2	7.8
48	Watanabe et al., 2011 [65]	2011	Japan	67	66 (26-87)	113	21.2	78.8	73.5	26.5	12.4	34.5	53.1	PNA/RT-PCR	31	NR	NR
49	Watanabe et al., 2013 [66]	2013	Japan	61	NR	5732	17.1	79.7	68.3	31.7	NR	NR	NR	Sequencing	37.6	79.5	20.5
50	Yari et al., 2020 [67]	2020	Iran	NR	NR	100	NR	NR	NR	NR	NR	NR	NR	Sequencing	29	72.4	20.7
51	Yip et al., 2013 [68]	2013	Malaysia	65	NR	44	51.2	48.8	68.3	31.7	7.3	82.9	9.8	Sequencing	25	72.7	18.2
52	Yoshino et al., 2015 [69]	2015	Japan	NR	NR	307	NR	NR	NR	NR	NR	NR	NR	Sequencing	37.8	80.2	18.1
53	Zahrani et al., 2014 [70]	2014	Saudi Arabia	63	56.7 (21-88)	150	16	84	78.7	21.3	NR	NR	NR	Sequencing	56	86.9	13
54	Zekri et al., 2019 [71]	2019	Saudi Arabia	NR	NR	45	NR	NR	NR	NR	NR	NR	NR	Sanger sequencing	51.1	NR	NR
55	Zekri et al., 2012 [72]	2012	Saudi Arabia	65	61 (21-80)	46	30	70	83	17	15	83	2	Sanger sequencing	32.6	86.7	13
56	Zhang et al., 2015 [73]	2015	China	59	62.1 (18-96)	1110	19.1	80.9	50.7	49.3	7.5	73.5	19	PCR-SS	45.4	79	21
57	Zhu et al., 2020 [74]	2020	China	70	NR	53	37.7	62.3	NR	NR	NR	NR	NR	Sequencing	47.2	NR	NR
58	Zhang et al., 2018 [75]	2018	China	62	64	813	52.4	47.5	45.5	54.5	4.1	73.7	17	Sequencing	42.6	NR	NR
59	Zihui Yong et al., 2020 [76]	2020	Singapore	53	62 (12-91)	363	0	100	77	23	NR	NR	NR	Sequencing	34.7	NR	NR

N: number; NR: not reported. ^∗^Percentage of all samples, age is presented in years (mean + SD/median (range/IQR)/range). HRMS: high resolution melting- (HRM-) sequencing; HRMA/P: high resolution melting assay/pyrosequencing; PNAM/PCR and PNAM/PCR/S: peptide nucleic acid-mediated polymerase chain reaction/sequencing; IHC: immunohistochemistry; SS: Sanger sequencing.

**Table 2:** Major characteristics of the prevalence of BRAF screening studies that were included in the meta-analysis.

S/N	Author	Year	Location	Male, *n* (%)	Age	Sample size	Tumour stage (early stage)^∗^	Tumour stage (late-stage)^∗^	Tumour location (colon)^∗^	Tumour location (rectum)^∗^	Tumour grade (poorly differentiated)^∗^	Tumour grade (moderately differentiated)^∗^	Tumour grade (well-differentiated)^∗^	Method	Total BRAF mutation (%)
1	Bagadi et al., 2012 [27]	2012	India	74	56 (23-93)	100	22.5	77.5	78	22	NR	NR	NR	Sequencing	17
2	Eachkoti et al., 2018 [77]	2018	India	49	51.2 ± 14.3	57	36.8	63.2	52.6	47.4	8.8	54.4	36.8	Sequencing	21.1
3	Fu et al., 2019 [78]	2019	China	60	60 (14-96)	5495	NR	NR	50.1	0.499	6.5	0.713	24.4	HRMS	2.8
4	Mohammadi Asl et al., 2014 [79]	2014	Iran	55	44.ynn (40-50)	80	NR	NR	NR	NR	NR	NR	NR	PCR-FFLP/S	46.3
5	He et al., 2020 [36]	2020	China	62	59 (26-83)	194	4.1	83	72.6	27.3	45.9	NR	NR	Sequencing	11.9
6	Hsieh et al., 2012 [37]	2012	Taiwan	NR	NR	182	NR	NR	NR	NR	NR	NR	NR	Sequencing	1.1
7	Jauhri et al., 2017a [39]	2017	India	61	NR	112	23.2	76.8	82.1	17.9	NR	NR	NR	Sequencing	7.1
8	Kaji et al., 2011 [41]	2011	Japan	35	68.9 + 9.8	98	NR	NR	NR	NR	NR	NR	NR	NR	0
9	Karbalaie Niya et al., 2016 [42]	2016	Iran	57	NR	1000	NR	NR	NR	NR	16.4	38.4	43.9	HRMS	0
10	Korphaisarn et al., 2019 [14]	2019	Thailand	57	64 (30-89)	108	24.1	75.9	82.4	17.6	6.5	86.1	4.6	PNAMPCR	1.9
11	Kwon et al., 2011 [43]	2011	South Korea	60	54 ± 12.33	92	0	100	56.5	43.5	15.2	67.4	12	PNAMPCR/S	3.3
12	Mohamed Suhaimi et al., 2015 [48]	2015	Singapore	55	58.5 (26-74)	44	50	40.9	54.5	45.5	NR	NR	NR	HRM-S	11.4
13	Nagakubo et al., 2019 [52]	2019	Japan	NR	NR	50	NR	NR	NR	NR	NR	NR	NR	SS	6
14	Nguyen et al., 2021 [53]	2021	Vietnam	56	59.94 ± 12.36	151	76.2	19.2	68.9	31.1	10.2	36.2	53.2	SS	2.6
15	Rozek et al., 2010 [80]	2010	Israel	5	NR	1297	NR	NR	NR	NR	NR	NR	NR	Sequencing	5
16	Saxena et al., 2018 [81]	2018	India	68	64 (26-90)	65	60	40	NR	NR	23.1	50.8	26.2	Immunohistochemistry	4.6
17	Shimada et al., 2018 [82]	2018	Japan	59	NR	111	NR	NR	NR	NR	NR	NR	NR	NGS	6.3
18	Siraj et al., 2014 [58]	2014	Saudi Arabia	51	NR	770	44.5	49.2	NR	NR	12.1	76.6	9.6	PCR/sequencing	2.2
19	Song et al., 2020 [59]	2020	China	61	NR	2356	44.8	45.2	NR	NR	18.7	NR	81.3	Sequencing	1
20	Taniguchi et al., 2020 [83]	2020	Japan	NR	66	325	NR	NR	NR	NR	NR	NR	NR	Sequencing	10.5
21	Taniguchi et al., 2018 [63]	2018	Japan	59	64 (26-89)	302	26.8	73.2	53	47	9.6	NR	NR	Sequencing	6
22	Vilkin et al., 2009 [84]	2009	Israel	47	67.6 ± 12.3	128	54.9	45.1	NR	NR	25.1	54.6	20.3	Sequencing	18.8
23	Wang et al., 2017 [85]	2017	China	NR	NR	1694	NR	NR	NR	NR	NR	NR	NR	Sequencing	4.2
24	Warsinggih et al., 2020 [86]	2020	Indonesia	46	56 ± 11.2	43	NR	NR	67.4	32.6	44.2	34.9	20.9	Sequencing	14
25	Yari et al., 2020 [67]	2020	Iran	NR	NR	100	NR	NR	NR	NR	NR	NR	NR	Sequencing	7
26	Yip et al., 2013 [68]	2013	Malaysia	65	NR	44	51.2	48.8	68.3	31.7	7.3	82.9	9.8	Sequencing	2.3
27	Zhang et al., 2020 [87]	2020	China	60	NR	480	18.8	81.3	44	56	21.5	NR	NR	Sequencing	4
28	Zhang et al., 2015 [73]	2015	China	59	62.1 (18-96)	1110	19.1	80.9	50.7	49.3	7.5	73.5	19	PCR-SS	3.1
29	Zhu et al., 2020 [74]	2020	China	70	NR	53	37.7	62.3	NR	NR	NR	NR	NR	Sequencing	11.3

N: number; NR: not reported. ^∗^Percentage of all samples, age is presented in years ((mean + SD/median (range/IQR)/range). HRMS: high resolution melting- (HRM-) sequencing; HRMA/P: high resolution melting assay/pyrosequencing; PNAM/PCR and PNAM/PCR/S: peptide nucleic acid-mediated polymerase chain reaction/sequencing; IHC: immunohistochemistry; SS: Sanger sequencing.

**Table 3:** Subgroup analysis. Prevalence of KRAS of patients with colorectal cancer stratified by study location of study.

	No. of Studies	Prevalence (%)	95% CI	*I* ^2^ (%)	*Q*	Heterogeneity test	
						DF	*P*
Location							
Iraq	1	48.0	0.346-0.617	NA	NA	NA	NA
Iran	12	32.2	0.293-0.353	45.35	20.129	11	0.044
Jordan	2	46.9	0.412-0.527	0	0.514	1	0.474
Saudi Arabia	9	35.7	0.265-0.460	91.95	99.408	8	0.001
Japan	8	40.1	0.355-0.448	77.04	30.494	7	0.001
India	3	34.0	0.237-0.461	83.77	12.320	2	0.002
Taiwan	3	33.4	0.284-0.387	0	0.672	2	0.715
China	7	39.9	0.361-0.439	91.21	68.226	6	0.001
Thailand	1	47.2	0.380-0.566	NA	NA	NA	NA
South Korea	3	34.3	0.246–0.456	87.18	15.600	2	0.001
Kazakhstan	1	44.9	0.396-0.503	NA	NA	NA	NA
Oman	1	23.5	0.176-0.306	NA	NA	NA	NA
Singapore	2	34.4	0.299-0.392	0	0.145	1	0.703
Pakistan	1	13.3	0.088-0.198	NA	NA	NA	NA
Vietnam	1	37.1	0.298-0.451	NA	NA	NA	NA
Indonesia	1	71.8	0.559-0.836	NA	NA	NA	NA
Sri Lanka	1	23.1	0.161-0.320	NA	NA	NA	NA
Israel	1	44.9	0.402-0.497	NA	NA	NA	NA
Malaysia	1	25.0	0.144-0.397	NA	NA	NA	NA
Overall	59	36.3	0.345-0.382	85.54	401.015	58	0.001
KRAS subgroup by period of study conduct							
2010 and below	19	32.3	0.288-0.360	90.78	187.902	18	0.001
After 2010	28	39.9	0.373-0.425	82.25	152.081	27	0.001
Early tumour stage^1^	27	30.3	0.224-0.395	96.11	768.164	26	0.001
Late tumour stage^2^	27	67.9	0.593-0.755	82.25	668.459	26	0.001
KRAS subgroup by tumour location							
Colon	26	61.2	0.551-0.670	92.78	346.249	25	0.001
Rectum	26	39.3	0.336-0.453	92.34	326.498	25	0.001
KRAS subgroup by tumour grading							
Poor	23	9.6	0.063-0.145	90.420	229.651	22	0.001
Moderate	23	52.1	0.429-0.612	94.777	421.176	22	0.001
Well	23	31.0	0.214-0.425	96.266	589.219	22	0.001

^1^Implies stages 1 and 2; ^2^implies stages 3 and 4.

**Table 4:** Subgroup analysis. Prevalence of BRAF gene mutation of patients with colorectal cancer stratified by study location.

Subgroup	No. of Studies	Prevalence (%)	95% CI	*I* ^2^ (%)	*Q*	Heterogeneity test	
						DF	*P*
India	4	11.7	0.062-0.210	73.69	11.401	3	0.010
China	7	4.0	0.025-0.063	93.42	91.205	6	0.001
Iran	3	4.7	0.004-0.403	96.16	52.129	2	0.001
Taiwan	1	1.1	0.003-0.043	NA	NA	NA	NA
Japan	5	6.9	0.044-0.107	55.86	9.062	4	0.060
Thailand	1	1.9	0.005-0.071	NA	NA	NA	NA
South Korea	1	3.3	0.011-0.096	NA	NA	NA	NA
Singapore	1	11.4	0.048-0.245	NA	NA	NA	NA
Vietnam	1	2.6	0.010-0.068	NA	NA	NA	NA
Israel	2	9.8	0.025-0.316	96.90	32.271	1	0.001
Saudi Arabia	1	2.2	0.014-0.035	NA	NA	NA	NA
Indonesia	1	14.0	0.064-0.278	NA	NA	NA	NA
Malaysia	1	2.3	0.003-0.144	NA	NA	NA	NA
Overall	29	5.6	0.039-0.080	94.00	466.942	28	0.001
BRAF subgroup by period of study conduct							
2010 and below	8	5.6	0.020-0.146	96.45	196.928	7	0.001
After 2010	17	5.4	0.037-0.077	91.27	183.302	16	0.001
BRAF subgroup by tumour stage							
Early tumour stage^1^	10	40.1	0.293-0.518	62.297	62.297	9	0.005
Late tumour stage^2^	10	59.9	0.482-0.707	95.59	62.297	9	0.005
BRAF subgroup by tumour location							
Colon	10	67.9	0.577-0.766	54.421	19.746	9	0.020
Rectum	10	32.1	0.234-0.423	54.421	19.746	9	0.020
BRAF subgroup by tumour grade							
Poor	11	30.4	0.189-0.450	88.066	83.794	10	0.001
Moderate	11	56.3	0.432-0.686	86.413	73.599	10	0.001
Well	11	10.2	0.056–0.179	69.996	33.329	10	0.001

^1^Implies stages 1 and 2; ^2^implies stages 3 and 4.

## Data Availability

All data accessed and analysed in this study are available in the article and its Supplementary Materials.

## Supplementary Materials

Supplementary Materials Supplementary Figure File SF1-20: search strategy, forest plot of the pooled prevalence of KRAS and BRAF in colorectal cancer CRC patients in Asia stratified by study location, period of study, tumour location, tumour stage, and tumour grade; JBI file: Joanna Briggs Institute (JBI) critical appraisal checklist for prevalence studies; PRISMA file: quality assessment of included studies.
