# Supplementary material for: A Systematic Review and Meta-analysis on the Occurrence of Biomarker Mutation in Colorectal Cancer among the Asian Population
Source: Biomed Res Int. 2022 Jun 23;2022:5824183. doi: 10.1155/2022/5824183 (PMC9246611; doi:10.1155/2022/5824183)
Supplement: Supplementary Materials — Supplementary Figure File SF1-20: search strategy, forest plot of the pooled prevalence of KRAS and BRAF in colorectal cancer CRC patients in Asia stratified by study location, period of study, tumour location, tumour stage, and tumour grade; JBI file: Joanna Briggs Institute (JBI) critical appraisal checklist for prevalence studies; PRISMA file: quality assessment of included studies. [file 5824183.f1.zip › Supplementary Figure SF.docx]

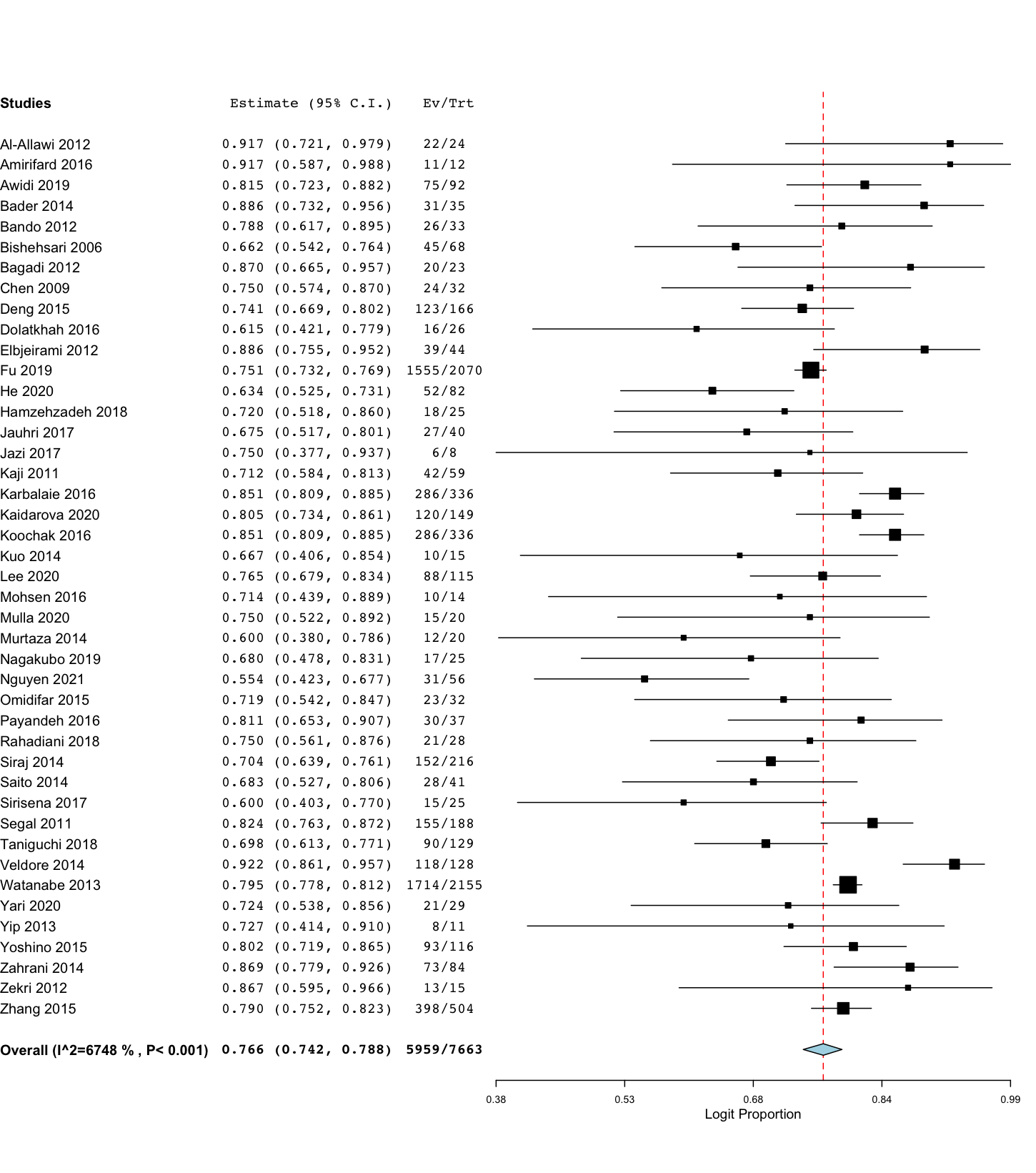
**Supplementary Figure SF1:** Forest plot for KRAS codon 12


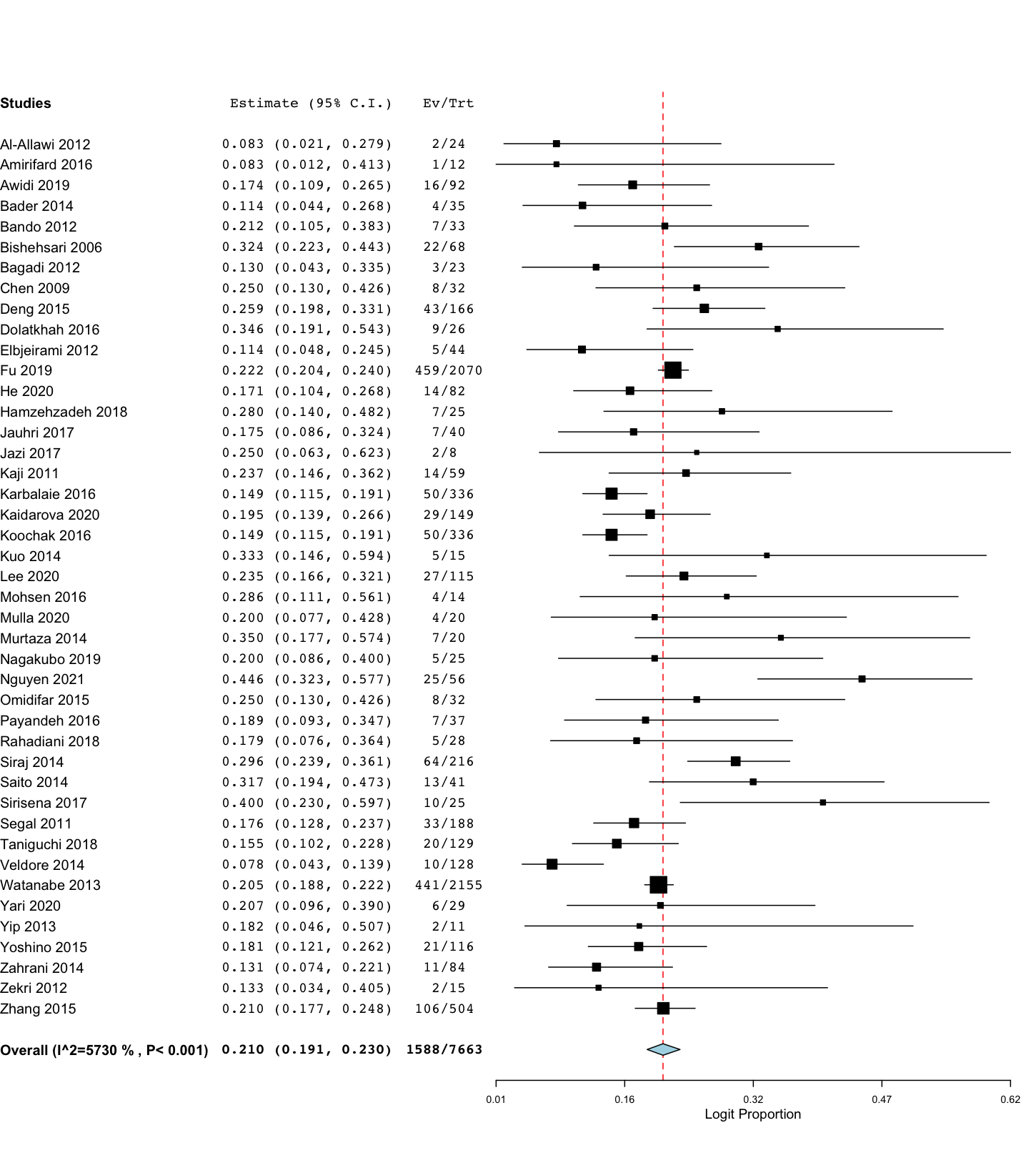


**Supplementary Figure SF2:** Forest plot for KRAS codon 13


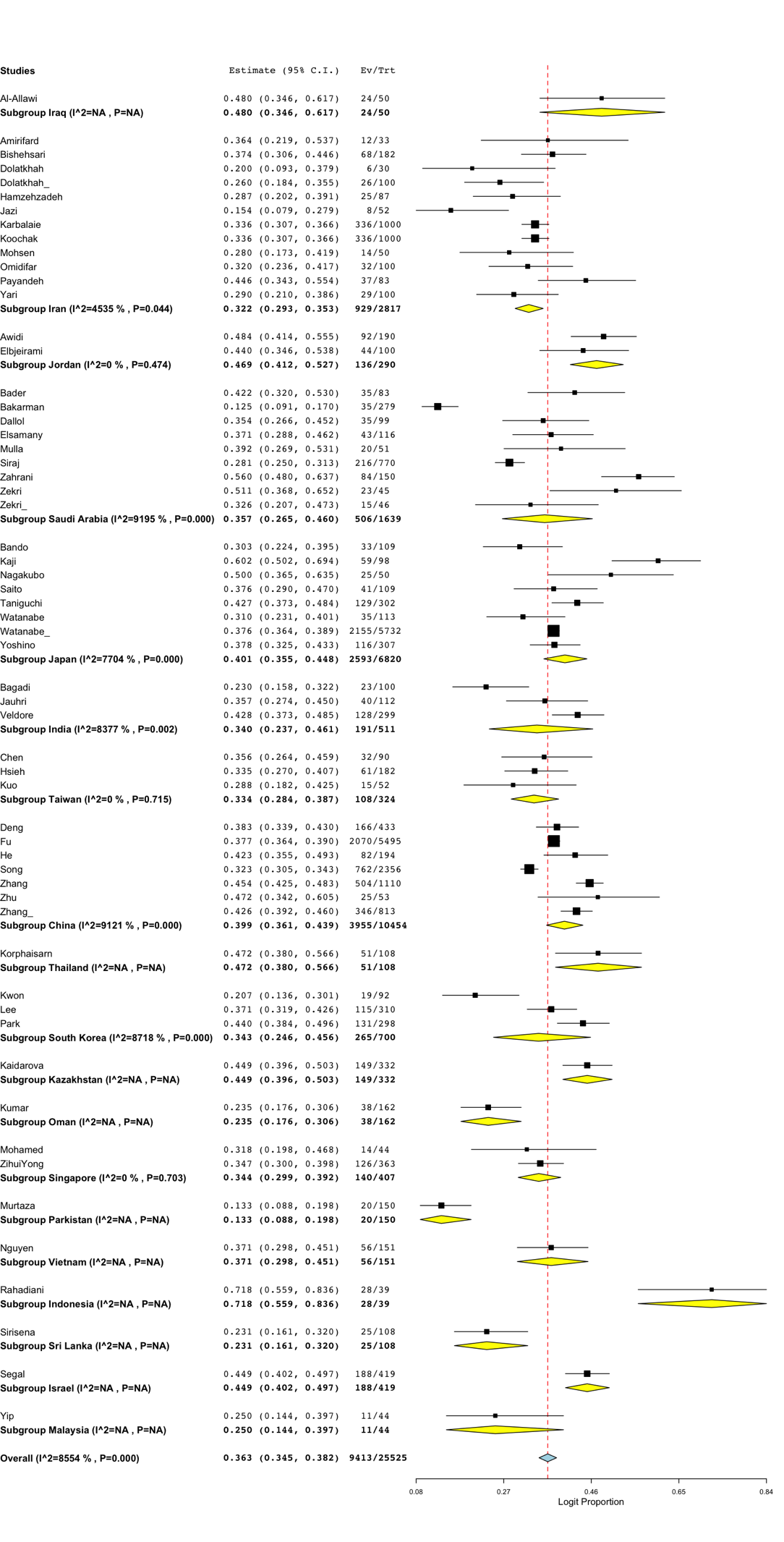


**Supplementary Figure SF3:** Forest plot for KRAS subgroup by country


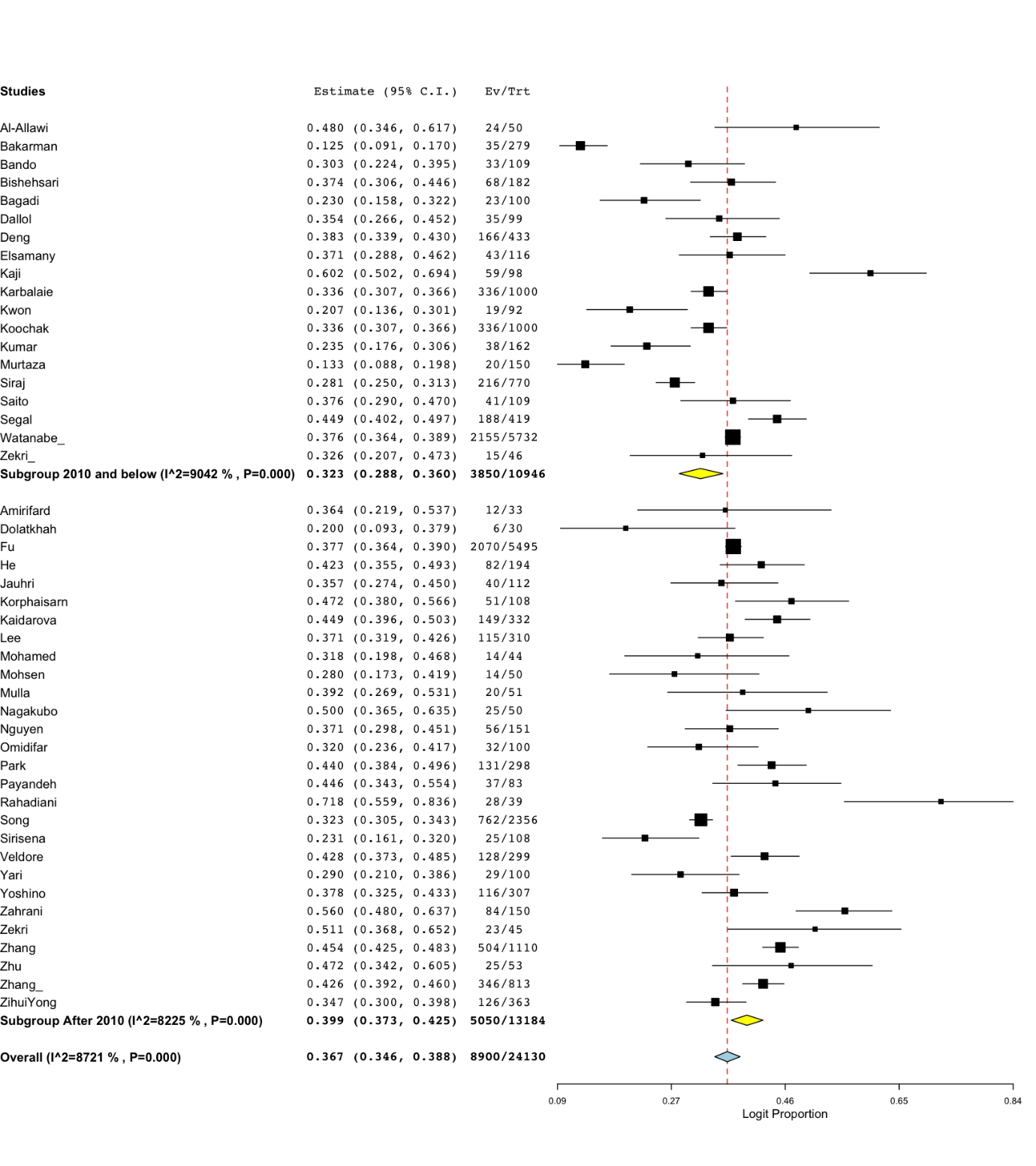


**Supplementary Figure SF4:** Forest plot for KRAS subgroup by period of study


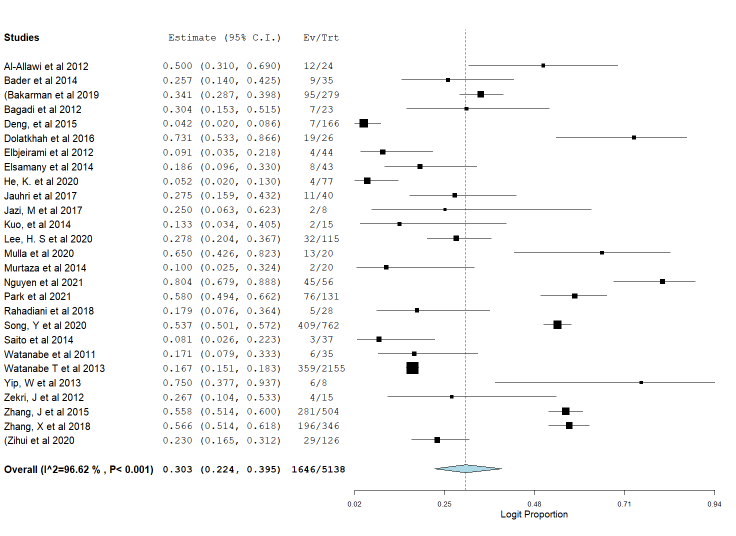


**Supplementary Figure SF5:** Forest plot for KRAS subgroup by early tumour stage (Stage 1&2)


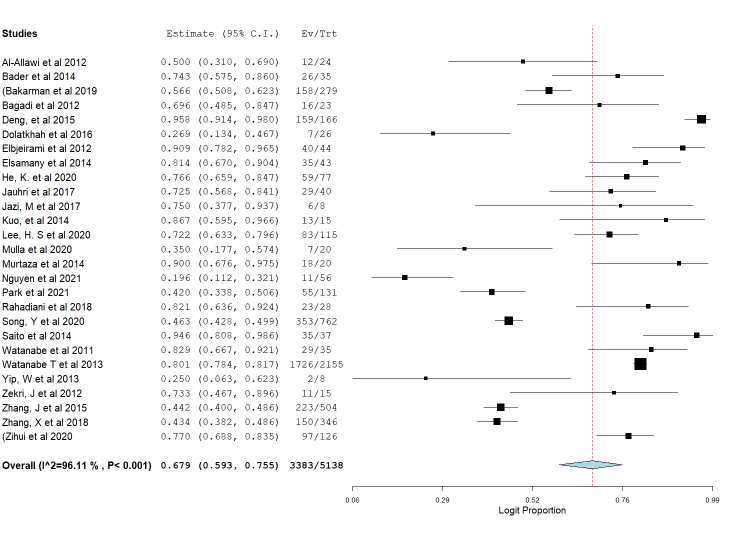


**Supplementary Figure SF6:** Forest plot for KRAS subgroup by late tumour stage (Stage 3&4)


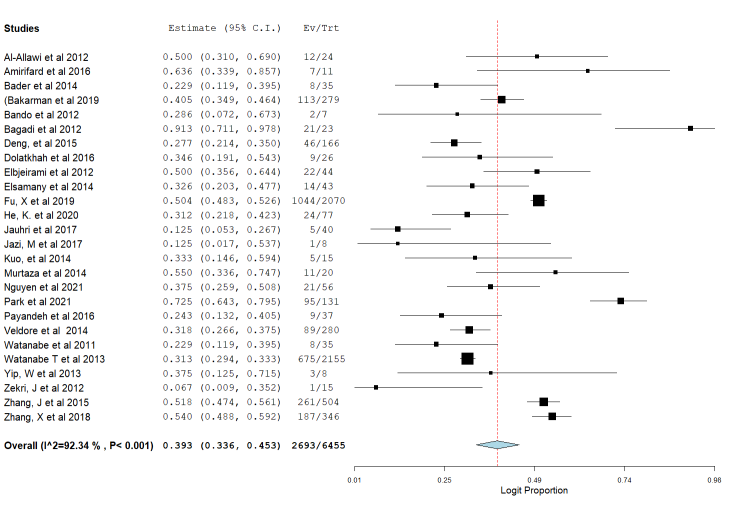


**Supplementary Figure SF7:** Forest plot for KRAS subgroup by tumour location (Rectum)


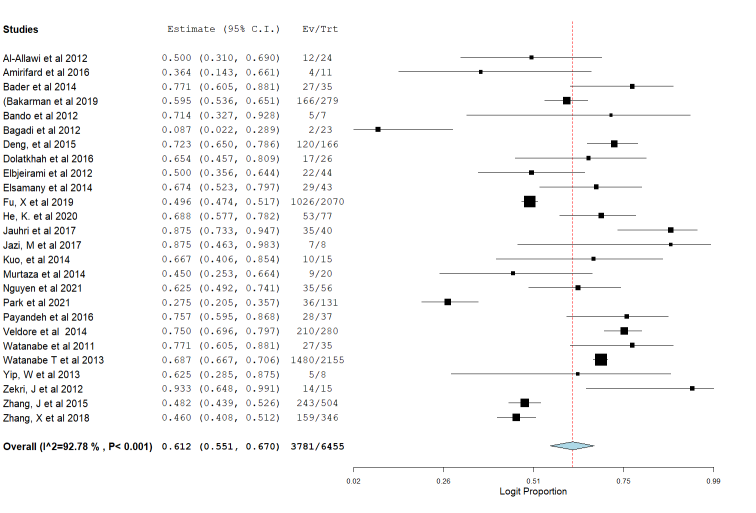


**Supplementary Figure SF8:** Forest plot for KRAS subgroup by tumour location (Colon)


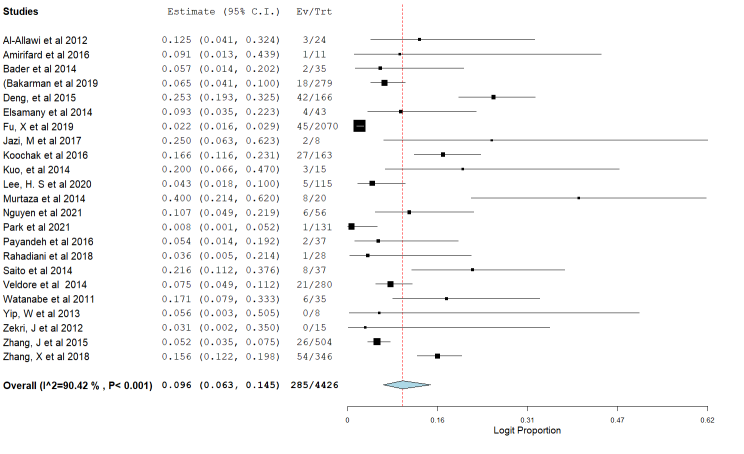


**Supplementary Figure SF9:** Forest plot for KRAS subgroup by tumour grade (Poor)


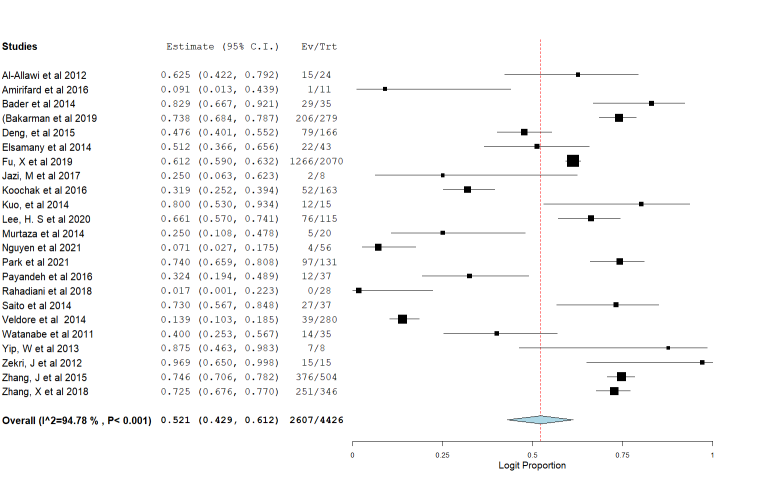


**Supplementary Figure SF10:** Forest plot for KRAS subgroup by tumour grade (Moderate)


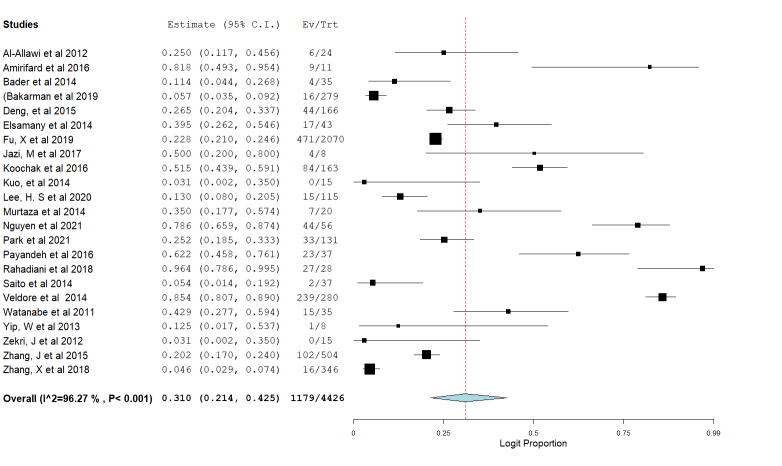


**Supplementary Figure SF11:** Forest plot for KRAS subgroup by tumour grade (Well)


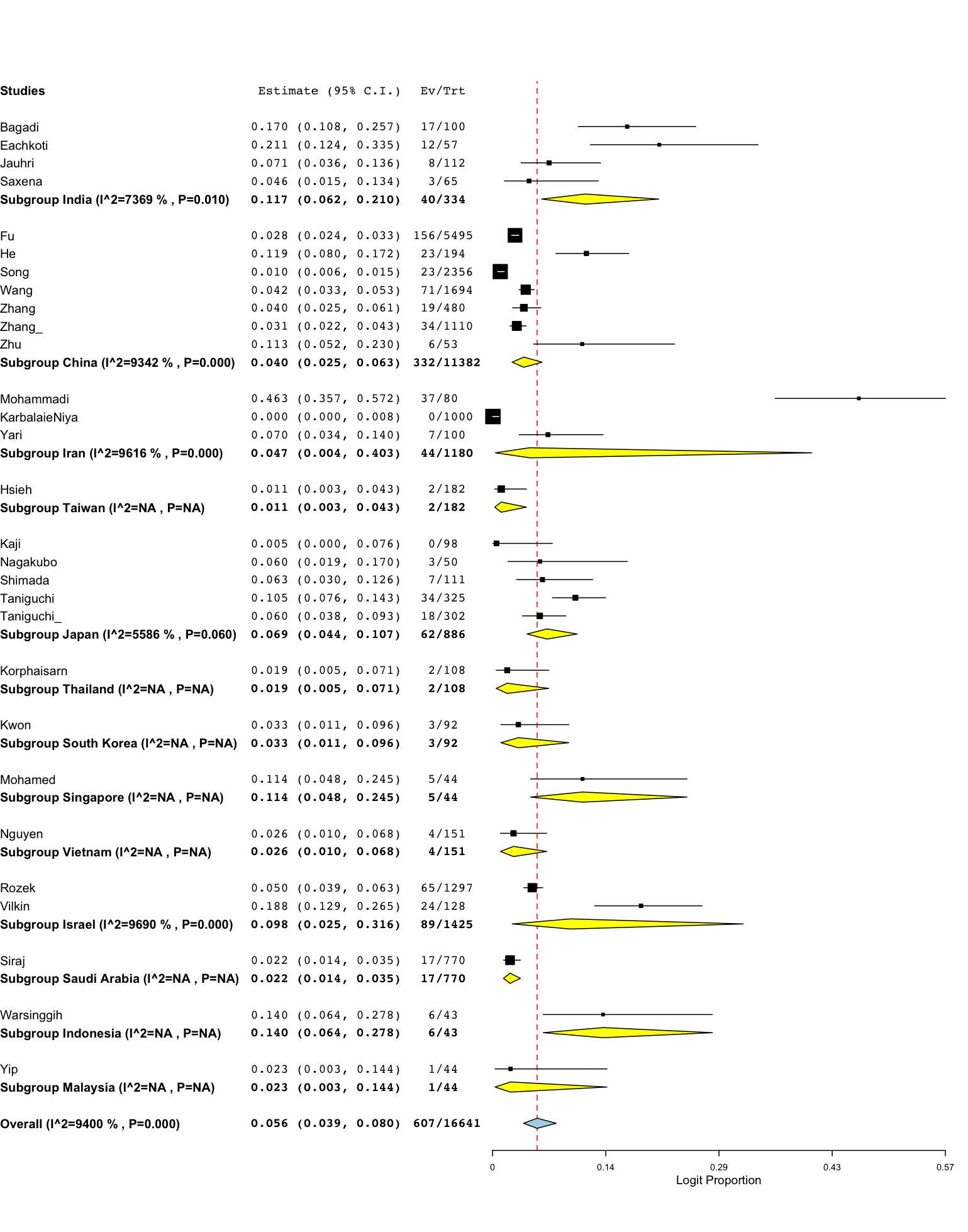


**Supplementary Figure SF12:** Forest plot for BRAF subgroup by country


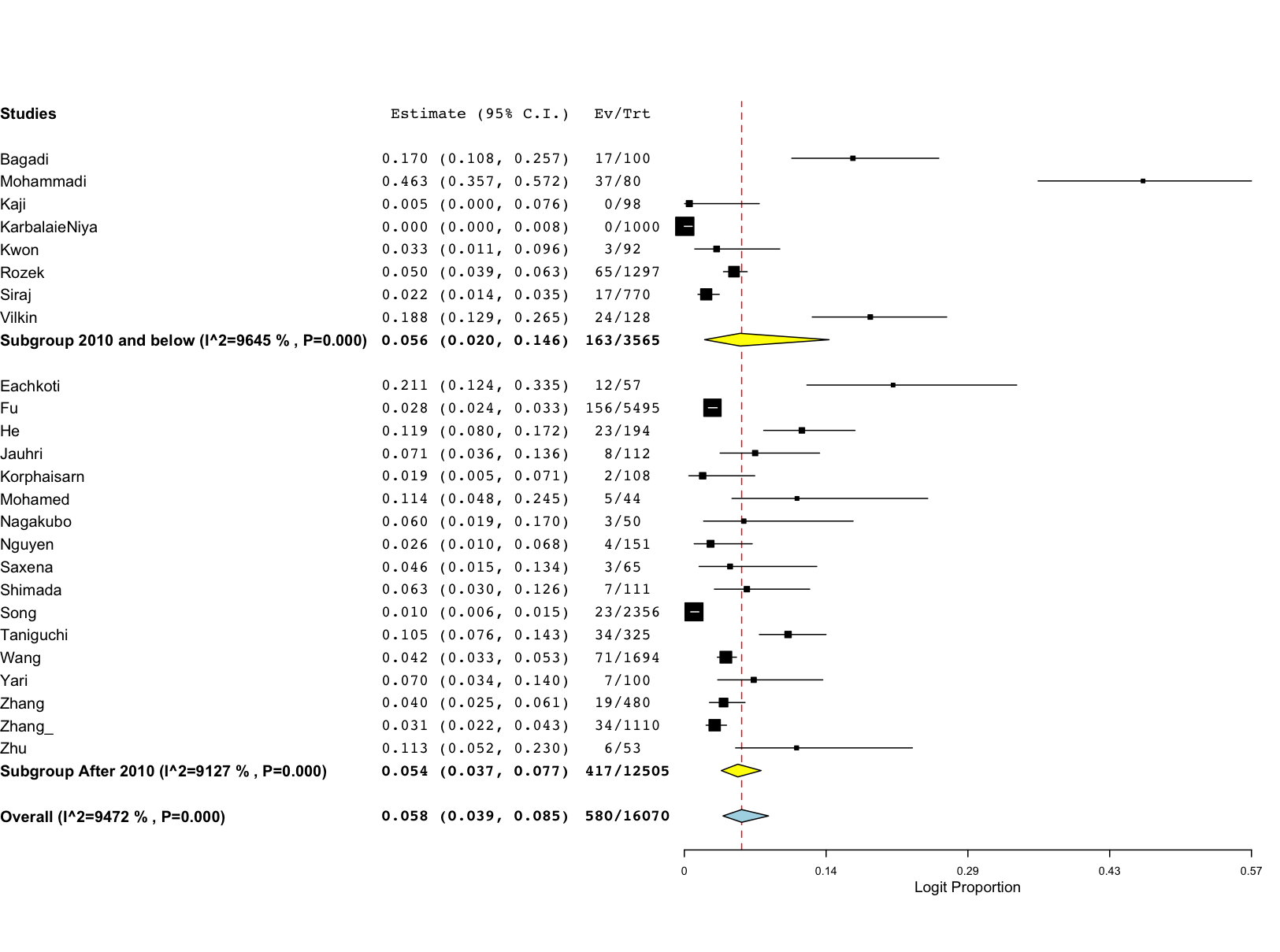


**Supplementary Figure SF13:** Forest plot for BRAF subgroup by year of sample collection


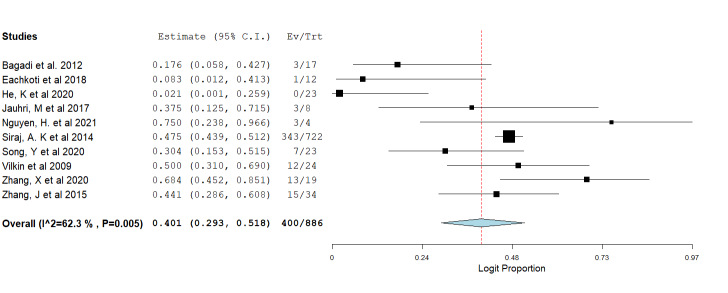
**Supplementary Figure SF14:** Forest plot for BRAF subgroup by early tumour stage (stage 1&2)


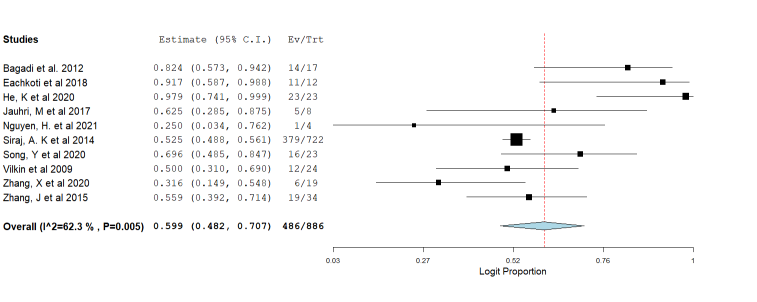


**Supplementary Figure SF15:** Forest plot for BRAF subgroup by late tumour stage (stage 3&4)


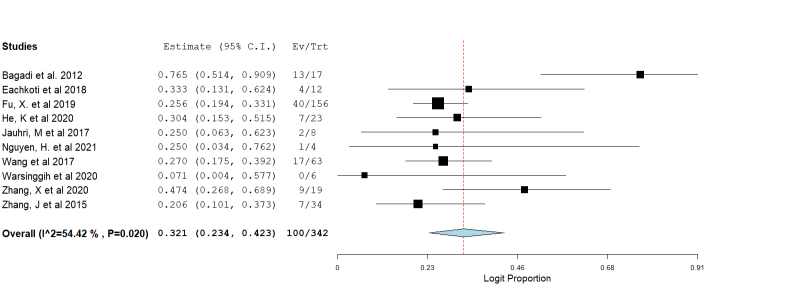


**Supplementary Figure SF16:** Forest plot for BRAF subgroup by tumour location (Rectum)


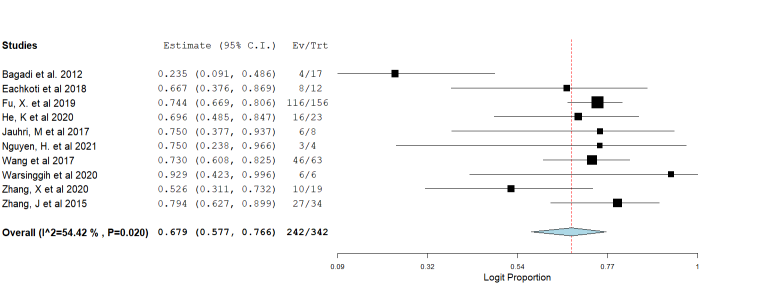
**Supplementary Figure SF17:** Forest plot for BRAF subgroup by tumour location (Colon)


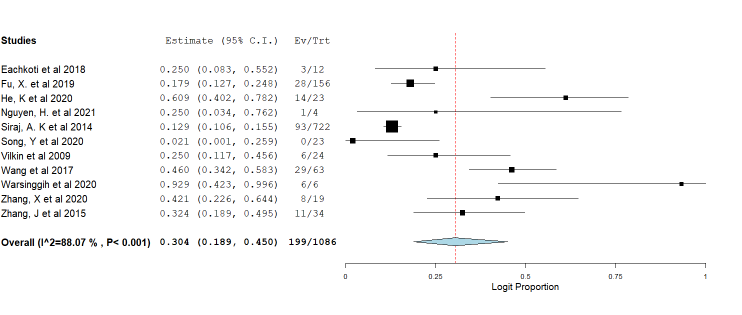


**Supplementary Figure SG18:** Forest plot for BRAF subgroup by tumour grade (Poor)


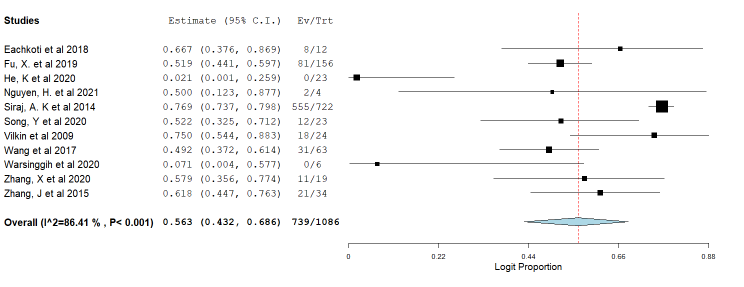


**Supplementary Figure SF19:** Forest plot for BRAF subgroup by tumour grade (Moderate)


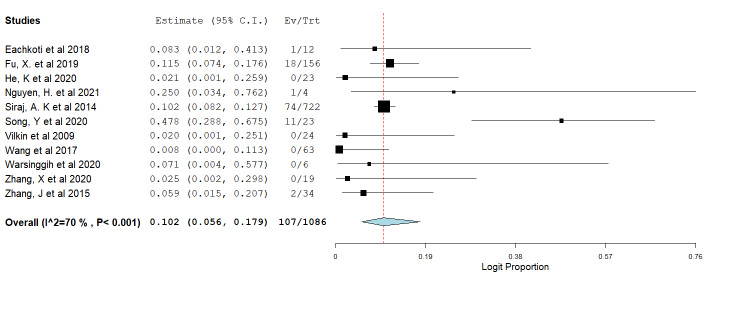


**Supplementary Figure SF20:** Forest plot for BRAF subgroup by tumour grade (Well)
