# Supplementary material for: A Systematic Review and Meta-analysis on the Occurrence of Biomarker Mutation in Colorectal Cancer among the Asian Population
Source: Biomed Res Int. 2022 Jun 23;2022:5824183. doi: 10.1155/2022/5824183 (PMC9246611; doi:10.1155/2022/5824183)
Supplement: Supplementary Materials — Supplementary Figure File SF1-20: search strategy, forest plot of the pooled prevalence of KRAS and BRAF in colorectal cancer CRC patients in Asia stratified by study location, period of study, tumour location, tumour stage, and tumour grade; JBI file: Joanna Briggs Institute (JBI) critical appraisal checklist for prevalence studies; PRISMA file: quality assessment of included studies. [file 5824183.f1.zip › Search Strategy file.docx]

**SEARCH STRATEGY**

PubMed (N = 188)

(("colorectal cancer"[Title/Abstract] OR "colon cancer"[Title/Abstract] OR "colorectal neoplasm"[Title/Abstract] OR "colorectal carcinoma"[Title/ Abstract] OR "colon tumour"[Title/Abstract] OR "colorectal tumour"[Title/ Abstract] OR "Rectum"[Title/Abstract] OR "metastatic colon cancer"[Title/ Abstract] OR "metastatic colorectal cancer"[Title/Abstract] OR "CRC"[Title/ Abstract] OR "mCRC"[Title/Abstract] OR "colon neoplasm"[Title/Abstract] OR "colon carcinoma"[Title/Abstract]) AND ("China"[Title/Abstract] OR "India"[Title/Abstract] OR "Indonesia"[Title/Abstract] OR "Pakistan"[Title/ Abstract] OR "Bangladesh"[Title/Abstract] OR "Japan"[Title/Abstract] OR "Philippines"[Title/Abstract] OR "Vietnam"[Title/Abstract] OR "turkey"[Title/ Abstract] OR "Iran"[Title/Abstract] OR "Thailand"[Title/Abstract] OR "Myanmar"[Title/Abstract] OR "south Korea"[Title/Abstract] OR "Iraq"[Title/ Abstract] OR "Afghanistan"[Title/Abstract] OR "Saudi Arabia"[Title/Abstract] OR "Uzbekistan"[Title/Abstract] OR "Malaysia"[Title/Abstract] OR "Yemen" [Title/Abstract] OR "Nepal"[Title/Abstract] OR "north Korea" [Title/Abstract] OR "Sri Lanka"[Title/Abstract] OR "Kazakhstan"[Title/ Abstract] OR "Syria" [Title/Abstract] OR "Cambodia"[Title/Abstract] OR "Jordan"[Title/Abstract] OR "Azerbaijan"[Title/Abstract] OR "united Arab emirate"[Title/Abstract] OR "Tajikistan"[Title/Abstract] OR "Israel"[Title/ Abstract] OR "Laos"[Title/ Abstract] OR "Lebanon"[Title/Abstract] OR "Kyrgyzstan"[Title/Abstract] OR "Turkmenistan"[Title/Abstract] OR "Singapore"[Title/Abstract] OR "Oman" [Title/Abstract] OR "Kuwait"[Title/ Abstract] OR "Georgia"[Title/ Abstract] OR "Mongolia"[Title/ Abstract] OR "Armenia"[Title/ Abstract] OR "Qatar"[Title/Abstract] OR "Bahrain"[Title/Abstract] OR "Timor-Leste"[Title/ Abstract] OR "Cyprus"[Title/Abstract] OR "Bhutan"[Title/Abstract] OR "Maldives"[Title/Abstract] OR "Brunei"[Title/Abstract] OR "Asia"[Title/Abstract])) AND ((((((("BRAF"[Title/Abstract]) OR ("B-RAF"[Title/Abstract])) OR ("cBRAF"[Title/Abstract])) OR ("c-BRAF"[Title/Abstract])) OR ("KRAS"[Title/Abstract] OR ("K-RAS"[Title/Abstract] OR ("c-KRAS"[Title/Abstract])

Scopus format (N = 217)

TITLE-ABS("colorectal cancer" OR "colon cancer" OR "colorectal neoplasm" OR "colorectal carcinoma" OR "colon tumour" OR "colorectal tumour" OR "Rectum" OR "metastatic colon cancer" OR "metastatic colorectal cancer" OR "CRC" OR "mCRC" OR "colon neoplasm" OR "colon carcinoma") AND TITLE-ABS("China" OR "India" OR "Indonesia" OR "Pakistan" OR "Bangladesh" OR "Japan" OR "Philippines" OR "Vietnam" OR "turkey" OR "Iran" OR "Thailand" OR "Myanmar" OR "south Korea" OR "Iraq" OR "Afghanistan" OR "Saudi Arabia" OR "Uzbekistan" OR "Malaysia" OR "Yemen" OR "Nepal" OR "north Korea" OR "Sri Lanka" OR "Kazakhstan" OR "Syria" OR "Cambodia" OR "Jordan" OR "Azerbaijan" OR "united Arab emirate" OR "Tajikistan" OR "Israel" OR "Laos" OR "Lebanon" OR "Kyrgyzstan" OR "Turkmenistan" OR "Singapore" OR "Oman" OR "Kuwait" OR "Georgia" OR "Mongolia" OR "Armenia" OR "Qatar" OR "Bahrain" OR "Timor-Leste" OR "Cyprus" OR "Bhutan" OR "Maldives" OR "Brunei" OR "Asia") AND TITLE-ABS("BRAF" OR "B-RAF" OR "cBRAF" OR "c-BRAF" OR "KRAS" OR "K-RAS" OR "c-KRAS")

Science Direct format (N = 241)

("colorectal cancer" OR "colon cancer" OR "metastatic colon cancer" OR "CRC" OR "Rectum”) ("BRAF" OR "B-RAF" OR "c-BRAF" OR "KRAS" OR "K-RAS")
