# Supplementary material for: A Systematic Review and Meta-analysis on the Occurrence of Biomarker Mutation in Colorectal Cancer among the Asian Population
Source: Biomed Res Int. 2022 Jun 23;2022:5824183. doi: 10.1155/2022/5824183 (PMC9246611; doi:10.1155/2022/5824183)
Supplement: Supplementary Materials — Supplementary Figure File SF1-20: search strategy, forest plot of the pooled prevalence of KRAS and BRAF in colorectal cancer CRC patients in Asia stratified by study location, period of study, tumour location, tumour stage, and tumour grade; JBI file: Joanna Briggs Institute (JBI) critical appraisal checklist for prevalence studies; PRISMA file: quality assessment of included studies. [file 5824183.f1.zip › JBI file.docx]

| **Study name** | | **Checklist*** | | | | | | | | | **Overall** |
| --- | --- | --- | --- | --- | --- | --- | --- | --- | --- | --- | --- |
|  |  | 1 | 2 | 3 | 4 | 5 | 6 | 7 | 8 | 9 |  |
| 1 | (Al-Allawi et al., 2012) | Yes | No | Yes | Yes | Yes | Yes | Yes | Yes | unclear | 7 |
| 2 | (Amirifard et al., 2016) | Yes | No | Yes | No | Yes | Yes | Yes | Yes | Yes | 7 |
| 3 | (Awidi et al., 2019) | Yes | No | Yes | Yes | Yes | Yes | Yes | Yes | Yes | 8 |
| 4 | (Bader and Ismail, 2014) | Yes | No | Yes | Yes | Yes | Yes | Yes | Yes | Yes | 8 |
| 5 | (Bakarman and AlGarni, 2019a) | Yes | Yes | Yes | Yes | Yes | Yes | Yes | Yes | Yes | 9 |
| 6 | (Bando et al., 2012) | Yes | No | Yes | Yes | Yes | Yes | Yes | Yes | Yes | 8 |
| 7 | (Bishehsari et al., 2006) | Yes | Yes | Yes | No | Yes | Yes | Yes | Yes | Yes | 8 |
| 8 | (Bagadi et al., 2012) | Yes | No | Yes | No | Yes | Yes | Yes | Yes | Yes | 7 |
| 9 | (Chen et al., 2009) | Yes | No | Yes | No | Yes | Yes | Yes | Yes | Yes | 8 |
| 10 | (Dallol et al., 2016) | Yes | No | Yes | Yes | Yes | Yes | Yes | Yes | Yes | 8 |
| 11 | (Deng et al., 2015) | Yes | No | Yes | Yes | Yes | Yes | Yes | Yes | Yes | 8 |
| 12 | (Dolatkhah et al., 2015) | Yes | No | Yes | No | Yes | Yes | Yes | Yes | Yes | 7 |
| 13 | (Dolatkhah et al., 2016) | Yes | No | Yes | No | Yes | Yes | Yes | Yes | Yes | 7 |
| 14 | (Elbjeirami and Sughayer, 2012) | Yes | No | Yes | No | Yes | Yes | Yes | Yes | Yes | 7 |
| 15 | (Elsamany et al., 2014) | Yes | No | Yes | Yes | Yes | Yes | Yes | Yes | Yes | 8 |
| 16 | (Fu et al., 2019) | Yes | No | Yes | Yes | Yes | Yes | Yes | Yes | Yes | 8 |
| 17 | (He et al., 2020) | Yes | No | Yes | Yes | Yes | Yes | Yes | Yes | Yes | 8 |
| 18 | (Hsieh et al., 2012) | Yes | No | Yes | NO | Yes | Yes | Yes | Yes | Yes | 7 |
| 19 | (Hamzehzadeh et al., 2018) | Yes | No | Yes | Yes | Yes | Yes | Yes | Yes | Unclear | 7 |
| 20 | (Jauhri et al., 2017) | Yes | No | Yes | Yes | Yes | Yes | Yes | Yes | Yes | 8 |
| 21 | (Jazi et al., 2017) | Yes | No | Yes | No | Yes | Yes | Yes | Yes | Yes | 7 |
| 22 | (Kaji et al., 2011) | Yes | No | Yes | Yes | Yes | Yes | Yes | Yes | Yes | 8 |
| 23 | (Karbalaie Niya et al., 2016) | Yes | Yes | Yes | Yes | Yes | Yes | Yes | Yes | Yes | 9 |
| 24 | (Korphaisarn et al., 2019) | Yes | No | Yes | Yes | Yes | Yes | Yes | Yes | Yes | 8 |
| 25 | (Kwon et al., 2011) | Yes | No | Yes | Yes | Yes | Yes | Yes | Yes | Yes | 8 |
| 26 | (Kaidarova et al., 2020) | Yes | No | Yes | Yes | Yes | Yes | Yes | Yes | Yes | 8 |
| 27 | (Koochak et al., 2016) | Yes | Yes | Yes | Yes | Yes | Yes | Yes | Yes | Yes | 9 |
| 28 | (Kumar et al., 2015) | Yes | No | Yes | Yes | Yes | Yes | Yes | Yes | Yes | 8 |
| 29 | (Kuo et al., 2014) | Yes | No | Yes | Yes | Yes | Yes | Yes | Yes | Yes | 8 |
| 30 | (Lee et al., 2020) | Yes | No | Yes | Yes | Yes | Yes | Yes | Yes | Yes | 8 |
| 31 | (Mohamed Suhaimi et al., 2015) | Yes | No | Yes | No | Yes | Yes | Yes | Yes | Yes | 7 |
| 32 | (Mohsen et al., 2016) | Yes | No | Yes | Yes | Yes | Yes | Yes | Yes | Yes | 8 |
| 33 | (Mulla et al., 2020) | Yes | No | Yes | Yes | Yes | Yes | Yes | Yes | Yes | 8 |
| 34 | (Murtaza et al., 2014) | Yes | No | Yes | Yes | Yes | Yes | Yes | Yes | Yes | 8 |
| 35 | (Nagakubo et al., 2019) | Yes | No | Yes | No | Yes | Yes | Yes | Yes | Yes | 7 |
| 36 | (Nguyen et al., 2021) | Yes | No | Yes | Yes | Yes | Yes | Yes | Yes | Yes | 8 |
| 37 | (Omidifar et al., 2015) | Yes | No | Yes | Yes | Yes | Yes | Yes | Yes | Yes | 8 |
| 38 | (Park et al., 2021) | Yes | No | Yes | Yes | Yes | Yes | Yes | Yes | Yes | 8 |
| 39 | (Payandeh et al., 2016) | Yes | No | Yes | Yes | Yes | Yes | Yes | Yes | Yes | 8 |
| 40 | (Rahadiani et al., 2018) | Yes | No | Yes | Yes | Yes | Yes | Yes | Yes | Yes | 8 |
| 41 | (Siraj et al., 2014) | Yes | No | Yes | Yes | Yes | Yes | Yes | Yes | Yes | 8 |
| 42 | (Song et al., 2020) | Yes | No | Yes | Yes | Yes | Yes | Yes | Yes | Yes | 8 |
| 43 | (Saito et al., 2014) | Yes | No | Yes | Yes | Yes | Yes | Yes | Yes | Yes | 8 |
| 44 | (Sirisena et al., 2017) | Yes | No | Yes | Yes | Yes | Yes | Yes | Yes | Yes | 8 |
| 45 | (Segal et al., 2011) | Yes | No | Yes | No | Yes | Yes | Yes | Yes | Yes | 7 |
| 46 | Taniguchi, H., et al. (2018) | Yes | No | Yes | Yes | Yes | Yes | Yes | Yes | Yes | 8 |
| 47 | (Veldore et al., 2014) | Yes | No | Yes | Yes | Yes | Yes | Yes | Yes | Yes | 8 |
| 48 | (Watanabe et al., 2011) | Yes | No | Yes | Yes | Yes | Yes | Yes | Yes | Yes | 8 |
| 49 | (Watanabe et al., 2013) | Yes | No | Yes | Yes | Yes | Yes | Yes | Yes | Yes | 8 |
| 50 | (Yari et al., 2020) | Yes | No | Yes | Yes | Yes | Yes | Yes | Yes | Yes | 8 |
| 51 | (Yip et al., 2013) | Yes | No | Yes | Yes | Yes | Yes | Yes | Yes | Yes | 8 |
| 52 | (Yoshino et al., 2015) | Yes | No | Yes | No | Yes | Yes | Yes | Yes | Yes | 7 |
| 53 | (Zahrani et al., 2014) | Yes | No | Yes | Yes | Yes | Yes | Yes | Yes | Yes | 8 |
| 54 | (Zekri et al., 2019) | Yes | No | Yes | No | Yes | Yes | Yes | Yes | Yes | 7 |
| 55 | (Zekri et al., 2012) | Yes | No | Yes | Yes | Yes | Yes | Yes | Yes | Yes | 8 |
| 56 | (Zhang et al., 2015) | Yes | No | Yes | Yes | Yes | Yes | Yes | Yes | Yes | 8 |
| 57 | (Zhu et al., 2020) | Yes | No | Yes | Yes | Yes | Yes | Yes | Yes | Yes | 7 |
| 58 | (Zhang et al., 2018) | Yes | No | Yes | Yes | Yes | Yes | Yes | Yes | Yes | 8 |
| 59 | (Zihui Yong et al., 2020) | Yes | No | Yes | Yes | Yes | Yes | Yes | Yes | Yes | 8 |
| 60 | (Bagadi et al., 2012) | Yes | No | Yes | Yes | Yes | Yes | Yes | Yes | Yes | 8 |
| 61 | (Eachkoti et al., 2018) | Yes | No | Yes | Yes | Yes | Yes | Yes | Yes | Yes | 8 |
| 62 | (Fu et al., 2019) | Yes | No | Yes | Yes | Yes | Yes | Yes | Yes | Yes | 8 |
| 63 | (Mohammadi Asl et al., 2014) | Yes | No | Yes | Yes | Yes | Yes | Yes | Yes | Yes | 8 |
| 64 | (He et al., 2020) | Yes | No | Yes | Yes | Yes | Yes | Yes | Yes | Yes | 8 |
| 65 | (Hsieh et al., 2012) | Yes | No | Yes | Yes | Yes | Yes | Yes | Yes | Yes | 8 |
| 66 | (Jauhri et al., 2017) | Yes | No | Yes | Yes | Yes | Yes | Yes | Yes | Yes | 8 |
| 67 | (Kaji et al., 2011) | Yes | No | Yes | Yes | Yes | Yes | Yes | Yes | Yes | 8 |
| 68 | (Karbalaie Niya et al., 2016) | Yes | No | Yes | Yes | Yes | Yes | Yes | Yes | Yes | 8 |
| 69 | (Korphaisarn et al., 2019) | Yes | No | Yes | Yes | Yes | Yes | Yes | Yes | Yes | 8 |
| 70 | (Kwon et al., 2011) | Yes | No | Yes | Yes | Yes | Yes | Yes | Yes | Yes | 8 |
| 71 | (Mohamed Suhaimi et al., 2015) | Yes | No | Yes | Yes | Yes | Yes | Yes | Yes | Yes | 8 |
| 72 | (Nagakubo et al., 2019) | Yes | No | Yes | Yes | Yes | Yes | Yes | Yes | Yes | 7 |
| 73 | (Nguyen et al., 2021) | Yes | No | Yes | Yes | Yes | Yes | Yes | Yes | Yes | 8 |
| 74 | (Rozek et al., 2010) | Yes | No | Yes | Yes | Yes | Yes | Yes | Yes | Yes | 8 |
| 75 | (Saxena et al., 2018) | Yes | No | Yes | Yes | Yes | Yes | Yes | Yes | Yes | 8 |
| 76 | (Shimada et al., 2018) | Yes | No | Yes | Yes | Yes | Yes | Yes | Yes | Yes | 7 |
| 77 | (Siraj et al., 2014) | Yes | No | Yes | Yes | Yes | Yes | Yes | Yes | Yes | 8 |
| 78 | (Song et al., 2020) | Yes | No | Yes | Yes | Yes | Yes | Yes | Yes | Yes | 8 |
| 79 | (Taniguchi et al., 2020) | Yes | No | Yes | Yes | Yes | Yes | Yes | Yes | Yes | 8 |
| 80 | (Taniguchi et al., 2018) | Yes | No | Yes | Yes | Yes | Yes | Yes | Yes | Yes | 8 |
| 81 | (Vilkin et al., 2009) | Yes | No | Yes | Yes | Yes | Yes | Yes | Yes | Yes | 7 |
| 82 | (Wang et al., 2017) | Yes | No | Yes | Yes | Yes | Yes | Yes | Yes | Yes | 8 |
| 83 | (Warsinggih et al., 2020) | Yes | No | Yes | Yes | Yes | Yes | Yes | Yes | Yes | 8 |
| 84 | (Yari et al., 2020) | Yes | No | Yes | Yes | Yes | Yes | Yes | Yes | Yes | 8 |
| 85 | (Yip et al., 2013) | Yes | No | Yes | Yes | Yes | Yes | Yes | Yes | Yes | 8 |
| 86 | (Zhang et al., 2020) | Yes | No | Yes | Yes | Yes | Yes | Yes | Yes | Yes | 8 |
| 87 | (Zhang et al., 2015) | Yes | No | Yes | Yes | Yes | Yes | Yes | Yes | Yes | 7 |
| 88 | (Zhu et al., 2020) | Yes | No | Yes | Yes | Yes | Yes | Yes | Yes | Yes | 8 |

**SUPPLEMENTARY FILE S3:** The quality of the 13 included studies

*** 1.** Appropriate sampling frame to address target population, **2.** Appropriate sampling way of study participants, **3.** Adequate sample size, **4.** Detail description of study participants and settings, **5.** Data analysis with sufficient coverage of identified sample, **6.** Use of valid methods to identify the condition, **7.** Standard, reliable way of measurement of condition for all participants, **8.** Availability of appropriate statistical analysis, **9.** Adequate response rate and management of low response rate

**Quality of included studies by JBI critical appraisal checklist for studies reporting prevalence data**
